# Resist diabetes: A randomized clinical trial for resistance training maintenance in adults with prediabetes

**DOI:** 10.1371/journal.pone.0172610

**Published:** 2017-02-23

**Authors:** Brenda M. Davy, Richard A. Winett, Jyoti Savla, Elaina L. Marinik, Mary Elizabeth Baugh, Kyle D. Flack, Tanya M. Halliday, Sarah A. Kelleher, Sheila G. Winett, David M. Williams, Soheir Boshra

**Affiliations:** 1 Department of Human Nutrition, Foods and Exercise, Virginia Tech, Blacksburg, Virginia, United States of America; 2 Department of Psychology, Virginia Tech, Blacksburg, Virginia, United States of America; 3 Department of Human Development, Virginia Tech, Blacksburg, Virginia, United States of America; 4 Department of Psychiatry and Behavioral Sciences, Duke University Medical Center, Durham, North Carolina, United States of America; 5 PCR, Inc, Blacksburg, Virginia, United States of America; 6 Department of Behavioral and Social Sciences, Brown University School of Public Health, Providence, Rhode Island, United States of America; 7 Carilion Clinic, Roanoke, Virginia, United States of America; Indiana University Richard M Fairbanks School of Public Health, UNITED STATES

## Abstract

**Objective:**

To determine whether a social cognitive theory (SCT)-based intervention improves resistance training (RT) maintenance and strength, and reduces prediabetes prevalence.

**Research design and methods:**

Sedentary, overweight/obese (BMI: 25–39.9 kg/m^2^) adults aged 50–69 (N = 170) with prediabetes participated in the 15-month trial. Participants completed a supervised 3-month RT (2×/wk) phase and were randomly assigned (N = 159) to one of two 6-month maintenance conditions: SCT or standard care. Participants continued RT at a self-selected facility. The final 6-month period involved no contact. Assessments occurred at baseline and months 3, 9, and 15. The SCT faded-contact intervention consisted of nine tailored transition (i.e., supervised training to training alone) and nine follow-up sessions. Standard care involved six generic follow-up sessions. Primary outcomes were prevalence of normoglycemia and muscular strength.

**Results:**

The retention rate was 76%. Four serious adverse events were reported. After 3 months of RT, 34% of participants were no longer prediabetic. This prevalence of normoglycemia was maintained through month 15 (30%), with no group difference. There was an 18% increase in the odds of being normoglycemic for each % increase in fat-free mass. Increases in muscular strength were evident at month 3 and maintained through month 15 (P<0.001), which represented improvements of 21% and 14% for chest and leg press, respectively. Results did not demonstrate a greater reduction in prediabetes prevalence in the SCT condition.

**Conclusions:**

Resistance training is an effective, maintainable strategy for reducing prediabetes prevalence and increasing muscular strength. Future research which promotes RT initiation and maintenance in clinical and community settings is warranted.

**Trial Registration:**

ClinicalTrials.gov NCT01112709.

## Introduction

The prevalence of diabetes among adults in the United States (US) is estimated to be 12.3%, and 25.9% among those aged 65 years or older [1]. Prediabetes, defined as impaired fasting glucose (IFG) or impaired glucose tolerance (IGT), affects more than one-third of US adults, and 51% of individuals aged 65 years or older [1]. Up to 70% of individuals with prediabetes may advance to type 2 diabetes [2]; within one year, the rate of progression is 5–10% [3].

The Community Preventive Task Force recommends intensive combined diet and physical activity promotion programs to reduce type 2 diabetes, with reversion to normoglycemia ranging from 20% to 52% depending upon program duration and intensity [4]. However, limited evidence is available to determine the optimal maintenance phase structure and the effectiveness of web-based programs targeting lifestyle change [4]. Clinical practice recommendations for diabetes prevention include lifestyle modification, specifically 5–10% weight loss and 30 minutes of moderate intensity physical activity per day [2, 5]. Yet weight reduction remains a challenge for most individuals, and a “portfolio of approaches” to prevent diabetes may be needed to maximize intervention reach and effectiveness [6].

Resistance training (RT) is increasingly recognized as an important treatment component for type 2 diabetes [5, 7, 8]. This mode of exercise is particularly beneficial for older, prediabetic adults given the loss of lean body mass and worsening of glucose tolerance with advancing age [9, 10]. Regular RT engagement (1–2 sessions per week) is associated with lower odds of impaired glucose metabolism [11] and all-cause mortality [12], and with improved physical functioning [13]. Despite these benefits, less than 10% of adults aged 65 years and older report engaging in muscle strengthening exercise two or more times per week [12].

Continuing the beneficial lifestyle outcomes evident in a supervised clinical setting to largely unsupervised community settings has been a challenge for diabetes prevention programs [14–16]. Furthermore, exercise adherence is rarely reported following supervised exercise intervention phases [17] in typical diabetes prevention programs. These issues also have been a focus of studies including individuals with type 2 diabetes, and were initially resistance training under supervision and then either trained at community facilities or at home [18, 19]. Some evidence of effectiveness for glycemic control was found, enhanced by theory-based behavior change procedures, though adherence to the established protocol varied [18, 19]. However, the standard protocol used in these studies involved training three times per week with between 24 to 32 sets per training session.

The Resist Diabetes trial was designed to assess if an RT protocol entailing training twice per week and involving 12 sets per session could reduce prediabetes prevalence and improve strength in a supervised clinical setting, and then be successfully translated and maintained without direct supervision in community settings by implementing a Social Cognitive Theory (SCT)-based maintenance intervention. Using results from this trial, we previously reported that the initiation of RT was associated with short-term changes in dietary intake (e.g., reduced intake of carbohydrates and total sugars [20], that short-term improvements in glucose tolerance with RT initiation may be limited to individuals with prediabetes who have IGT (vs isolated IFG) [21], RT adherence and cost estimates of the Standard and SCT-based intervention conditions [22], The current article presents the main trial outcomes. The primary objective of this trial was to determine whether an SCT-based intervention improves RT maintenance and muscular strength, and reduces prediabetes prevalence among older adults with prediabetes. It was hypothesized that SCT-based intervention will produce better outcomes than the Standard Care follow-up at 9-month and 15-month assessments.

## Materials and methods

### Study design

**Fig 1:**
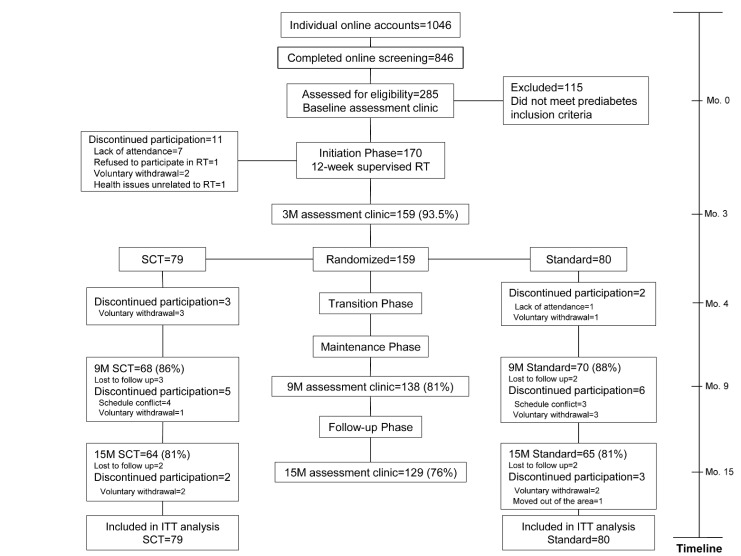
Resist Diabetes study consort diagram. Abbreviations: RT = resistance training; 3M = 3-month; SCT = social cognitive theory group; 9M = 9-month; 15M = 15-month; ITT = intent-to-treat.

Resist Diabetes was a 15-month randomized controlled trial including 170 men and women aged 50–69 years (N = 170) with prediabetes, defined as exhibiting either IFG (fasting glucose = 95–125 mg/dl) and/or IGT (2-hour glucose = 140–199 mg/dl) [23], and who met all other inclusion criteria (described below). Following screening and baseline testing (Fig 1), participants first completed a 3-month initiation phase. Resistance training sessions were completed two times per week on nonconsecutive days, and were supervised by an American College of Sports Medicine-certified Personal Trainer in a laboratory/gym. The protocol conformed to recommended guidelines [24] and included the following exercises on Nautilus equipment: leg press, leg extension, seated leg curl, calf raise, chest press, lat pulldown, row, shoulder press, seated dip, lower back, abdominal crunch and rotary torso. Participants performed one set of all 12 exercises at moderate resistance for 8–12 repetitions (3-second concentric, 3-second eccentric contractions) with good form and a high degree of effort to concentric failure. To progress to the next phase of the study, participants were required to attend at least 17 of the 24 scheduled RT sessions (70% minimum adherence).

After the initiation phase, participants (N = 159; 94% retention) were randomly assigned (by ELM) to one of two RT transition and maintenance conditions for 6 months: 1) a Social Cognitive Theory (SCT)-based intervention delivered over nine transition sessions and nine brief maintenance sessions using interactive, self-regulation procedures (e.g., goal setting, monitoring, reporting, feedback, planning, problem solving) with tailored in-person and web-based feedback, 2) a standard, usual care condition consisting of four transition and two brief maintenance sessions with SCT content (e.g., didactic instruction in problem solving) and generic web-based tracking of RT sessions [22]. The study principal investigators (BMD and RAW), statistician co-investigator (JSS), and participants were blinded to group assignment. Participants transitioned from supervised training to training alone at a self-selected community facility during the maintenance phase and paid associated membership fees. In both conditions, contact ended after 6 months, but participants were expected to continue RT at their respective facilities and all participants continued to have access to the web-based RT tracking system. Assessments occurred at baseline and months 3 (post-initiation), 9 (post-maintenance intervention), and 15 (after 6 months of no contact). A detailed description of study procedures has been published [25].

### Participants

Men and women aged 50–69 years were recruited from Roanoke, Virginia and its surrounding area using newspaper, workplace, and church advertisements and direct mailings between January 2011 and September 2012, and all follow-up assessments were completed by January 14 (trial ended as sample size was attained). Individuals were directed to a study information website, and if interested, they completed an online screening questionnaire (Fig 1). Internet access was required for participation as some intervention components were delivered online. Eligible individuals were apparently healthy (no known presence of heart disease), with no cardiovascular symptoms (e.g., chest discomfort, dizziness, shortness of breath). Inclusion criteria included a sedentary lifestyle (defined as moderate PA <120 min/week or vigorous PA <60 minutes/week), overweight or obese weight status (BMI 25–39.9 kg/m^2^), and not having engaged in RT in the past 12 months. Individuals who appeared eligible following the online screening were required to obtain medical clearance from their personal physician and were scheduled for baseline testing to determine prediabetes status. Only those meeting prediabetes criteria were eligible to participate.

Exclusion criteria were as follows: current smokers, cardiovascular disease diagnosis, pulmonary, liver or kidney disease, uncontrolled hypertension (BP>160/100 mmHg), diabetes or use of diabetes medications, conditions precluding RT such as major orthopedic injuries or musculoskeletal disabilities, and short-term use of any medications known to influence metabolism (e.g., beta blockers) or body weight (e.g., thyroid replacement, antidepressants). Individuals taking commonly prescribed medications (e.g., hormone replacement therapy) were eligible for participation if they had been on a stable dose of the medication for greater than one year. Individuals with hypertension whose blood pressure (BP) was adequately controlled (i.e., <140/<90 mm Hg) with antihypertensive medications were permitted to participate. The protocol was approved by the institutional review board (IRB) of Virginia Tech, and all participants gave written informed consent prior to enrollment.

### Assessments

At each assessment period, participants completed online surveys to determine physical activity level [26] and health beliefs related to RT [27] prior to each scheduled laboratory assessment. Laboratory testing took place over two days. The first day of testing included a detailed health and medical history, assessment of height, weight, body mass index (BMI), and BP. Height was measured using a wall-mounted stadiometer and body mass was measured using a digital scale (Healthometer ProPlus™). Body mass index (BMI) was calculated as weight (kg)/height (m)^2^. Blood pressure was measured according to recommended guidelines [28] using an automated device (Dinamap Procare Model 9300, GE HealthCare, Milwaukee, WI). The mean of two consecutive readings was used.

Fasting glucose and glucose tolerance were assessed using a 2-hr oral glucose tolerance test (OGTT). After a baseline fasting glucose sample was collected, participants consumed an 8-ounce 75 g glucose beverage (Fisherbrand, Fisher Scientific, Hanover Park, IL) within the first 5 min of the test. Additional samples were obtained at minutes 10, 20, 30, 60, 90 and 120. Glucose concentrations (mg/dl) were determined using a YSI 2700 Select glucose analyzer (YSI Life Sciences, Yellow Springs, OH). Insulin and C-peptide concentrations were analyzed using commercially-available assay kits (ELISA, ALPCO; IMMULITE, Siemens). HOMA was calculated to assess insulin resistance [29]. Glucose, insulin and C-peptide area-under-the-curve (AUC) were determined using the trapezoidal method [30].

Adherence was determined using a time-line follow-back approach at the 9- and 15-month assessment time points. On a printed calendar, participants noted each day of RT within the past 30 days [25]. Dietary intake was assessed using three 24-hour dietary recalls. The first recall was obtained in-person by a trained research dietitian, and the second and third recalls were completed via phone by the same dietitian in the two-week period following the testing session. Printed food diagrams were used to assist in portion size estimation. Recalls were analyzed using the Nutrition Data System for Research software (NDS-R 2010, University of Minnesota, Minneapolis, MN).

The second day of testing, included assessments of waist circumference, body composition (dual-energy x-ray absorptiometry), and strength tests (3-repetition maximum [RM]), at the laboratory/gym. Waist circumference was measured using a Gulick tape measure at the level of the umbilicus and recorded as the mean of two measurements. Body fat percent, absolute fat mass and fat-free mass was assessed using dual-energy X-ray absorptiometry (DXA; GE Lunar Prodigy, Madison, WI). Strength change on the chest press and leg press resistance machines was assessed during a three-repetition maximum (3RM) test following ACSM guidelines [24].

### Adverse events

Participants were instructed to immediately contact a member of the study staff by phone or email if a serious adverse event occurred; these were documented and reviewed by the study Medical Director to determine the appropriate course of action (e.g., discontinue training, medical follow-up).

### Sample size and power calculations

With a minimum sample size of 110, our study was powered to detect significant group differences (80%) in change over the four assessment points for achievement of normal FPG, 2-hr plasma glucose concentration and increases in strength, assuming a maximum difference between the control and treatment group to be 15–20%, comparable to Cohen’s *d* of 0.6 [31]. To allow for a 35% attrition rate, we recruited a sample of 170 participants to ensure that we had complete data on 110 participants at all four assessment points of the study [25].

### Statistical analysis

At month 3, a stratified random assignment procedure (generated by JSS) was used with sex and responsiveness (i.e., initial mean strength gains on chest press and leg press of <15%, 15% to <35%, or ≥35%) as randomization variables. No significant group or sex differences were found in participant characteristics at the time of randomization [25]. All analyses were conducted in STATA (version 14; StataCorp) using intention-to-treat and with 2-sided tests of α = .05. Linear mixed effects models were estimated to compare the outcomes between the two groups at baseline, month 3, 9, and 15. Random class effects were used to control for repeated measurements. The fixed effects parameters were group (SCT vs. Standard), month of assessment, and the interaction of group × time. Contrasts were used to estimate and test the Bonferroni-adjusted within-group differences. Secondary outcomes were analyzed using the same approach. Results are presented as the difference in regression coefficients, along with an overall test of interaction effect. For the proportion of participants who reverted to normoglycemia [5], we used a repeated measures generalized estimation model with *xtgee* procedure in STATA. Similar to the linear mixed models, this model included a random intercept to account for repeated measurements within participants, as well as fixed effects for group, time, and group × time interaction. The *xtgee* model controlled for sex, prediabetes phenotype at baseline and change in lean body mass percent over the three study phases. The results are presented as population-averaged odds ratios.

## Results

Baseline and month 3 (i.e., at randomization) participant characteristics are provided in **Table 1.** Participants were predominantly non-Hispanic, white, college-educated, and female. Mean BMI was in the obese range, and mean waist circumference was indicative of visceral obesity [32]. Almost half of participants were taking antihypertensive medications, and one-third were using statins (Table 1). One participant in the Standard group was prescribed metformin at month 3 by her personal physician. Of the antihypertensive medications used by participants, one has been reported to increase type 2 diabetes risk (i.e., hydrochlorothiazide[HCTZ]) [33]. Six individuals in each group were taking HCTZ. With regard to prediabetes phenotype, most participants were categorized as either isolated IFG or combined IFG/IGT. Upon randomization at month 3, there were no group differences in participant characteristics [25].

**Table 1:** Resist Diabetes: Participant characteristics at Baseline and at Randomization (Month 3).

	At Baseline (n = 170)	At Randomization	
		Standard (N = 80)	SCT (N = 79)
Age (years)	59.5 ±5.4	59.7 ±5.1	59.6 ±5.6
Sex			
Female	124 (73%)	57 (71%)	58 (73%)
Male	46 (27%)	23 (29%)	21 (27%)
Weight (kg)	93.31 ±13.82	93.74 ±14.62	92.85 ±13.47
BMI (kg/m^2^)	32.95 ±3.77	33.02 ±3.87	32.99 ±3.93
Blood Pressure (BP)			
Systolic BP (mmHg)	131 ±15	130 ±13	127 ±13
Diastolic BP (mmHg)	76 ±9	74 ±9	73 ±8
Body composition			
Fat percent (%)	43.83 ±6.95	43.24 ±6.71	43.07 ±6.91
Fat mass (kg)	40.61 ±8.36	40.10 ±8.57	39.62 ±8.07
Fat-free percent (%)	56.16 ±6.96	56.76 ±6.71	56.93 ±6.91
Fat-free mass (kg)	52.09 ±10.37	52.72 ±10.77	52.72 ±10.69
Waist Circumference (cm)	109.08 ±10.31	108.90 ±10.63	107.56 ±10.67
Strength			
Chest press 3RM (kg)	33.64 ±11.56	43.49 ±15.10	42.14 ±14.63
Leg press 3RM (kg)	140.99 ±35.90	167.23 ±37.63	165.44 ±41.20
Blood measures			
Fasting glucose (mg/dL)	102 ± 8	100 ±10	102 ±10
2hr glucose (mg/dL)	142 ± 36	136 ±37	133 ±35
Fasting insulin (uIU/mL)	15.2 ± 12.5	16.6 ±12.4	16.9 ±10.7
2hr insulin (uIU/mL)	139.2 ±122.8	140.6 ±122.9	136.6 ±116.0
HOMA-IR	3.83 ±3.28	4.17 ±3.29	4.34 ±2.83
Fasting C-peptide (ng/mL)	3.99 ±2.11	3.89 ±1.87	3.89 ±1.55
2hr C-peptide (ng/mL)	16.14 ±8.42	17.16 ±7.65	16.56 ±7.16
Prediabetes criteria			
Normal	0 (0%)	28 (35%)	27 (34%)
IFG	81 (48%)	21 (26%)	23 (29%)
IGT	21 (12%)	9 (11%)	9 (11%)
Both IFG & IGT	68 (40%)	22 (28%)	20 (25%)
Race			
White	160 (94%)	75 (94%)	74 (94%)
Black	9 (5%)	5 (6%)	4 (5%)
Other	1 (1%)	0 (0%)	1 (1%)
Ethnicity			
Hispanic	2 (1%)	0 (0%)	2 (3%)
Non-Hispanic	168 (99%)	80 (100%)	77 (97%)
Education level			
High school	6 (4%)	3 (4%)	1 (1%)
College (partial or completed)	111 (65%)	52 (65%)	52 (66%)
Grad/professional degree	53 (31%)	25 (31%)	26 (33%)
Medications			
Antihypertensives	82 (48%)	43 (54%)	37 (47%)
Statins	52 (31%)	19 (24%)	30 (38%)
Antidepressants	41 (24%)	19 (24%)	17 (22%)
Thyroid medications	35 (21%)	10 (13%)	22 (28%)
HRT	14 (8%)	8 (10%)	6 (8%)
Blood thinners	6 (4%)	4 (5%)	2 (3%)
Inhalers	5 (3%)	4 (5%)	0 (0%)
Eye disease medications	1 (1%)	1 (1%)	0 (0%)
Glucocorticoids	1 (1%)	0 (0%)	1 (1%)
Insulin-sensitizing medication	0 (0%)	1 (1%)	0 (0%)

Variables expressed as means±SD or frequency (%).

Abbreviations used: BMI, body mass index; RM, repetition maximum; HOMA-IR, Homeostatic model assessment of insulin resistance; IFG = impaired fasting glucose; IGT = impaired glucose tolerance; HRT = hormone replacement therapy.

SI conversion: To convert glucose to mmol/L, multiply by 0.0555; insulin to pmol/L, multiply by 6.945; C-peptide to nmol/L, multiply by 0.331.

The overall retention rate was 76% (**Fig 1**). Adherence to the twice weekly RT sessions during the 3-month Initiation Phase was 91% (i.e., 22 of 24 sessions completed) [34]. Self-reported adherence among those present at assessment sessions, using the time-line follow-back calendars completed at month 9, was 78% and 72% in the SCT and Standard groups, respectively. At month 15, adherence was 53% in both groups. There were no significant group differences in adherence. Including non-completers, and assuming no RT adherence among those individuals, adherence at 15 months was 42% (SCT) and 44% (Standard) [22].

Changes in primary and secondary outcomes over the study period are presented in **Tables 2**and **3**. No changes were noted in fasting glucose concentrations. However, glucose tolerance improved in the Standard group in the first 3 months of the trial, which was maintained at 15 months (11 mg/dl reduction) (p<0.05). No group differences over time were detected in fasting or 2-hour insulin concentrations, insulin and glucose AUC, HOMA-IR, or fasting C-peptide. A group difference over time was noted in 2-hr C-peptide concentrations (p = 0.05), with significant increases occurring in the SCT group from baseline to month 15. Eleven of 129 participants (Standard, 4; SCT, 7) were classified as diabetic according to fasting or 2-hour glucose concentrations at month 15.

**Table 2:** Changes in diabetes-related outcomes, strength, body weight and composition, blood pressure, physical activity and dietary intake during the 15-month Resist Diabetes trial.^A^

Study Group	Study Period Mean (SD) Score				Within-Group Difference Point Estimate (Bonferroni 95% CI)			p-value Overall Group x Time Interaction
	Baseline	3m	9m	15m	Baseline to 15 M	Baseline to 3 M	3M to 15M	
Fasting glucose (mg/dL) (n = 159, Obs = 585)								
SCT	102	102	104	103	0.13	0.05	0.08	0.75
	(8)	(10)	(13)	(14)	(-2.77 to 3.03)	(-2.64 to 2.75)	(-2.82 to 2.98)	
Standard	101	100	101	101	-0.25	-1.61	1.36	
	(9)	(10)	(11)	(10)	(-3.13 to 2.63)	(-4.29 to 1.07)	(-1.52 to 4.24)	
2hr glucose (mg/dL) (n = 159, Obs = 580)								
SCT	140	133	143	142	1.49	-6.86	8.35	0.004
	(38)	(35)	(45)	(45)	(-8.61 to 11.59)	(-16.18 to 2.45)	(-1.76 to 18.47)	
Standard	149	136	138	140	-11.09*	-12.97**	1.88	
	(33)	(37)	(36)	(37)	(-20.99 to -1.18)	(-22.18 to -3.75)	(-8.02 to 11.79)	
Glucose AUC (n = 109, Obs = 375)								
SCT	18911	19077	20033	18963	136.75	53.09	83.65	0.11
	(3132)	(3084)	(4343)	(3487)	(-901.13 to 1174.63)	(-884.46 to 990.64)	(-963.58 to 1130.89)	
Standard	19070	17950	18561	18488	-501.63	-1112.29**	610.66	
	(2865)	(3173)	(2969)	(3168)	(-1487.63 to 484.36)	(-2000.56 to -224.02)	(-337.44 to 1598.76)	
Fasting insulin (uIU/mL) (n = 157, Obs = 532)								
SCT	14.2	16.9	19.7	17.3	4.22**	3.05	1.17	0.09
	(11.3)	(10.7)	(13.2)	(8.2)	(0.78 to 7.66)	(-0.21 to 6.32)	(-2.05 to 4.39)	
Standard	16.4	16.6	17.0	17.4	1.68	0.89	0.79	
	(14.1)	(12.4)	(9.2)	(9.8)	(-1.62 to 4.97)	(-2.24 to 4.03)	(-2.36 to 3.93)	
2hr insulin (uIU/mL) (n = 157, Obs = 528)								
SCT	126.6	136.6	131.3	124.8	-13.13	2.37	-15.50	0.99
	(109.5)	(116.0)	(94.1)	(84.9)	(-41.48 to 15.22)	(-24.74 to 29.49)	(-41.69 to 10.69)	
Standard	158.6	140.6	127.4	133.3	-14.23	-12.80	-1.43	
	(138.5)	(122.9)	(89.7)	(107.6)	(-41.25 to 12.78)	(-38.46 to 12.86)	(-27.06 to 24.19)	
Insulin AUC (n = 106, Obs = 327)								
SCT	9848	10530	10030	10135	-247.60	264.38	-511.98	0.76
	(6343)	(6831)	(6641)	(7292)	(-2073.18 to 1577.98)	(-1444.05 to 1972.82)	(-2098.56 to 1074.59)	
Standard	10809	9931	10186	10125	-834.14	-1040.43	206.29	
	(7380)	(7719)	(6869)	(6701)	(-2434.36 to 766.07)	(-2548.18 to 467.32)	(-1288.87 to 1701.44)	
HOMA-IR (mg/dL) (n = 157, Obs = 532)								
SCT	3.6	4.3	5.1	4.5	1.20**	0.84†	0.36	0.06
	(2.9)	(2.8)	(3.6)	(2.3)	(0.26 to 2.13)	(-0.04 to 1.72)	(-0.52 to 1.23)	
Standard	4.2	4.2	4.3	4.4	0.39	0.16	0.23	
	(3.8)	(3.3)	(2.5)	(2.6)	(-0.50 to 1.28)	(-0.69 to 1.01)	(-0.62 to 1.08)	
Fasting C-peptide (ng/mL) (n = 159, Obs = 584)								
SCT	4.1	3.9	3.9	3.8	-0.22	-0.22	0.01	0.34
	(2.2)	(1.5)	(1.8)	(1.6)	(-0.67 to 0.24)	(-0.64 to 0.19)	(-0.45 to 0.46)	
Standard	3.9	3.9	3.8	3.9	0.01	0.01	0.00	
	(2.0)	(1.9)	(1.5)	(1.5)	(-0.44 to 0.45)	(-0.40 to 0.42)	(-0.45 to 0.44)	
2hr C-peptide (ng/mL) (n = 159, Obs = 581)								
SCT	15.0	16.6	18.1	17.8	2.60**	1.69†	0.90	0.05
	(7.1)	(7.2)	(6.8)	(7.5)	(0.74 to 4.45)	(-0.03 to 3.42)	(-0.94 to 2.75)	
Standard	17.5	17.2	18.1	18.4	0.81	-0.36	1.17	
	(9.6)	(7.7)	(6.4)	(6.7)	(-1.02 to 2.64)	(-2.06 to 1.33)	(-0.65 to 3.00)	
C-peptide AUC (n = 109, Obs = 375)								
SCT	966	1068	1195	1243	260.36**	100.02	160.34*	0.08
	(513)	(504)	(458)	(497)	(113.70 to 407.01)	(-33.65 to 233.69)	(13.33 to 307.34)	
Standard	1124	1156	1274	1274	132.63	25.70	106.94	
	(537)	(428)	(369)	(424)	(-5.90 to 271.16)	(-100.96 to 152.36)	(-32.82 to 246.69)	
Chest press 3RM (kg) (n = 159, Obs = 569)								
SCT	33.50	42.14	42.47	43.44	10.65**	8.90**	1.75	0.23
	(11.75)	(14.63)	(17.00)	(17.74)	(8.41 to 12.90)	(6.90 to 10.90)	(-0.48 to 3.99)	
Standard	34.02	43.49	43.43	42.43	9.23**	9.39**	-0.15	
	(11.66)	(15.10)	(15.29)	(14.72)	(7.06 to 11.41)	(7.39 to 11.38)	(-2.33 to 2.02)	
Leg press 3RM (n = 159, Obs = 560)								
SCT	139.29	165.44	163.54	164.22	26.83**	26.89**	-0.05	0.21
	(38.27)	(41.20)	(40.44)	(38.52)	(19.92 to 33.75)	(20.73 to 33.05)	(-6.98 to 6.87)	
Standard	142.94	167.23	167.04	164.93	21.74**	24.30**	-2.56	
	(34.13)	(37.63)	(38.38)	(40.93)	(15.06 to 28.41)	(18.25 to 30.34)	(-9.23 to 4.11)	
Weight (kg) (n = 159, Obs = 585)								
SCT	92.82	92.85	92.14	91.65	-0.58	0.03	-0.61	0.77
	(13.30)	(13.47)	(13.97)	(13.99)	(-1.61 to 0.46)	(-0.92 to 0.99)	(-1.64 to 0.42)	
Standard	93.89	93.74	92.77	92.74	-0.82	-0.15	-0.68	
	(14.21)	(14.62)	(13.47)	(13.51)	(-1.84 to 0.20)	(-1.09 to 0.80)	(-1.70 to 0.34)	
BMI (kg/m2) (n = 159, Obs = 585)								
SCT	32.98	32.99	32.75	32.62	-0.22	0.01	-0.23	0.90
	(3.85)	(3.93)	(4.00)	(3.94)	(-0.58 to 0.15)	(-0.33 to 0.35)	(-0.60 to 0.14)	
Standard	33.07	33.02	32.79	32.63	-0.27	-0.06	-0.22	
	(3.71)	(3.87)	(3.92)	(4.03)	(-0.64 to 0.09)	(-0.39 to 0.28)	(-0.58 to 0.15)	
Body fat percent (%) (n = 159, Obs = 581)								
SCT	43.73	43.07	42.98	42.75	-0.47	-0.66**	0.19	0.73
	(6.89)	(6.91)	(6.64)	(6.57)	(-0.99 to 0.06)	(-1.14 to -0.18)	(-0.34 to 0.72)	
Standard	43.82	43.24	42.99	42.85	-0.54*	-0.58**	0.04	
	(6.79)	(6.71)	(6.98)	(7.08)	(-1.06 to -0.02)	(-1.06 to -0.10)	(-0.48 to 0.56)	
Fat mass (kg) (n = 159, Obs = 581)								
SCT	40.35	39.62	39.21	39.07	-0.75	-0.73†	-0.02	0.85
	(7.81)	(8.07)	(8.10)	(8.22)	(-1.57 to 0.07)	(-1.47 to -0.01)	(-0.84 to 0.80)	
Standard	40.79	40.10	39.55	39.25	-0.86*	-0.69	-0.17	
	(8.43)	(8.57)	(8.76)	(8.87)	(-1.66 to -0.06)	(-1.43 to 0.05)	(-0.97 to 0.63)	
Fat-free mass percent (n = 159, Obs = 581)								
SCT	56.27	56.93	57.02	57.21	0.43	0.66**	-0.23	0.66
	(6.89)	(6.91)	(6.64)	(6.58)	(-0.10 to 0.95)	(0.18 to 1.13)	(-0.75 to 0.30)	
Standard	56.17	56.76	56.98	57.13	0.53*	0.59**	-0.06	
	(6.80)	(6.71)	(6.98)	(7.09)	(0.01 to 1.04)	(0.11 to 1.06)	(-0.58 to 0.45)	
Fat-free mass (kg) (n = 159, Obs = 581)								
SCT	52.01	52.72	52.12	51.92	-0.33	0.71**	-1.04**	0.27
	(10.29)	(10.69)	(10.12)	(10.27)	(-0.99 to 0.32)	(0.11 to 1.30)	(-1.69 to -0.38)	
Standard	52.43	52.72	52.41	52.27	-0.05	0.29	-0.34	
	(10.57)	(10.77)	(9.98)	(9.87)	(-0.69 to 0.59)	(-0.30 to 0.88)	(-0.98 to 0.30)	
Waist Circumference (cm) (n = 159, Obs = 580)								
SCT	108.83	107.56	106.47	106.49	-2.86**	-1.27*	-1.59**	0.17
	(10.36)	(10.67)	(10.33)	(10.77)	(-4.22 to -1.49)	(-2.50 to -0.03)	(-2.96 to -0.22)	
Standard	109.75	108.90	107.66	107.50	-1.97**	-0.86	-1.12	
	(10.19)	(10.63)	(10.77)	(10.79)	(-3.30 to -0.64)	(-2.08 to 0.37)	(-2.45 to 0.22)	
Systolic Blood Pressure (mmHg) (n = 159, Obs = 580)								
SCT	131	127	130	131	0.49	-3.82†	4.31†	0.32
	(17)	(13)	(15)	(15)	(-3.80 to 4.78)	(-7.81 to 0.16)	(-0.002 to 8.62)	
Standard	132	130	131	132	-1.08	-1.59	0.51	
	(13)	(13)	(15)	(14)	(-5.34 to 3.17)	(-5.54 to 2.35)	(-3.74 to 4.77)	
Diastolic Blood Pressure (mmHg) (n = 159, Obs = 580)								
SCT	75	73	73	73	-1.70	-2.71**	1.01	0.80
	(8)	(8)	(8)	(9)	(-3.80 to 0.40)	(-4.65 to -0.76)	(-1.10 to 3.11)	
Standard	76	74	75	75	-1.32	-1.79†	0.47	
	(9)	(9)	(10)	(9)	(-3.39 to 0.76)	(-3.71 to -0.13)	(-1.61 to 2.55)	
MET/hr/wk (other than RT) (n = 157, Obs = 507)								
SCT	11.78	13.07	15.11	15.97	4.80**	1.02	3.78†	0.59
	(10.87)	(8.50)	(13.35)	(10.99)	(0.64 to 8.97)	(-2.60 to 4.64)	(-0.36 to 7.93)	
Standard	11.70	13.15	15.86	14.28	3.40	1.78	1.62	
	(9.20)	(11.03)	(14.28)	(17.07)	(-0.61 to 7.41)	(-1.72 to 5.28)	(-2.36 to 5.60)	
Total MET/hr/wk (including RT) (n = 159, Obs = 526)								
SCT	11.96	21.34	23.73	20.89	10.13***	9.54***	0.59	0.66
	(10.89)	(9.38)	(14.89)	(11.71)	(5.62 to 14.63)	(5.64 to 13.44)	(-3.81 to 4.99)	
Standard	11.89	21.25	23.52	19.43	8.99***	10.04***	-1.05	
	(9.17)	(12.32)	(16.00)	(18.61)	(4.70 to 13.28)	(6.24 to 13.84)	(-5.27 to 3.16)	
Energy (kcals) (n = 159, Obs = 583)								
SCT	1762.31	1735.56	1690.11	1739.74	-16.23	-21.71	5.47	0.10
	(494.43)	(474.28)	(456.68)	(527.35)	(-155.60 to 123.13)	(-150.56 to 107.15)	(-133.33 to 144.27)	
Standard	1850.42	1751.23	1762.99	1720.87	-160.31**	-99.19	-61.12	
	(517.33)	(451.89)	(452.07)	(471.45)	(-297.31 to -23.31)	(-226.72 to 28.34)	(-198.12 to 75.88)	
Carbohydrate (%) (n = 159, Obs = 583)								
SCT	44.19	43.06	42.23	43.25	-1.00	-1.10	0.10	0.37
	(7.44)	(8.13)	(9.15)	(9.65)	(-3.83 to 1.83)	(-3.72 to 1.52)	(-2.72 to 2.92)	
Standard	42.76	42.61	41.74	42.85	0.37	-0.15	0.52	
	(8.38)	(8.75)	(9.62)	(10.68)	(-2.42 to 3.15)	(-2.75 to 2.44)	(-2.26 to 3.30)	
Fat (%) (n = 159, Obs = 583)								
SCT	36.00	36.05	36.88	36.44	0.74	0.08	0.66	0.08
	(6.99)	(6.60)	(7.19)	(7.50)	(-1.72 to 3.21)	(-2.21 to 2.38)	(-1.80 to 3.12)	
Standard	37.39	37.13	38.02	35.63	-1.75	-0.27	-1.48	
	(6.92)	(6.38)	(7.02)	(7.17)	(-4.17 to 0.68)	(-2.54 to 2.00)	(-3.90 to 0.95)	
Protein (%) (n = 159, Obs = 583)								
SCT	18.17	18.95	18.55	17.96	-0.36	0.71	-1.07	0.05
	(4.45)	(4.30)	(4.86)	(4.44)	(-1.99 to 1.27)	(-0.80 to 2.23)	(-2.69 to 0.55)	
Standard	17.88	18.21	18.37	19.28	1.35	0.33	1.02	
	(4.14)	(4.28)	(4.43)	(5.98)	(-0.26 to 2.95)	(-1.17 to 1.83)	(-0.58 to 2.62)	
Fiber (g) (n = 159, Obs = 583)								
SCT	18.60	17.87	18.52	17.69	-1.22	-0.72	-0.49	0.73
	(7.59)	(6.97)	(6.93)	(6.68)	(-3.19 to 0.75)	(-2.55 to 1.10)	(-2.46 to 1.47)	
Standard	18.66	17.65	18.03	18.33	-0.73	-1.01	0.28	
	(6.75)	(5.93)	(6.68)	(6.29)	(-2.67 to 1.21)	(-2.81 to 0.79)	(-1.66 to 2.21)	
Added sugar (g) (n = 159, Obs = 583)								
SCT	53.67	50.52	44.41	49.98	-2.12	-2.88	0.76	0.73
	(30.74)	(31.09)	(21.15)	(27.67)	(-11.15 to 6.92)	(-11.24 to 5.48)	(-8.24 to 9.76)	
Standard	51.63	46.90	48.93	49.34	-2.55	-4.73	2.18	
	(30.27)	(30.84)	(27.05)	(33.64)	(-11.44 to 6.33)	(-13.01 to 3.54)	(-6.70 to 11.06)	

Abbreviations used: AUC, area under the curve; BMI, body mass index; MET, metabolic equivalents; M, month; RM, repetition maximum; SCT, Social Cognitive Theory.

^A^ Data are presented for 159 participants, except for the AUC data which included a subset of 109 participants.

† *p <* 0.10

* *p* < 0.05

** *p* < 0.01

*** *p* < 0.001.

**Table 3:** Odds of achieving normoglycemia among Resist Diabetes trial participants.^a^

Parameter	Coefficient (SE)	OR	95% CI	Interpretation
Phase				
SCT Group				
Initiation	Ref	1.00		
Maintenance (M 9)	0.11 (0.31)	1.12	(0.61, 2.04)	
No-Contact (M 15)	-0.11 (0.33)	0.90	(0.47, 1.70)	
STD Group				
Initiation	-0.03 (0.35)	0.97	(0.49, 1.94)	
Maintenance (M 9)	-0.31 (0.37)	0.74	(0.35, 1.53)	
No Contact (M 15)	-0.26 (0.38)	0.77	(0.36, 1.63)	
Δ in Lean Mass Percent^b^	0.18 (0.08) *	1.19	(1.02, 1.39)	The odds of reverting to normoglycemia are higher for participants with an increase in lean body mass percentage.
Gender				
Male	Ref	1.00		
Female	-0.37 (0.30)	0.69	(0.38, 1.24)	
Prediabetes Phenotype				
IFG and IGT	Ref	1.00		
IFG	1.27 (0.32) **	3.58	(1.93, 6.63)	Participants with isolated IFG and isolated IGT have higher odds of reverting to normoglycemia compared to those with both IFG & IGT.
IGT	1.59 (0.43) **	4.92	(2.13, 11.34)	
Intercept	-1.34 (0.39) **	0.26	(0.12, 0.56)	

N_Initiation_ = 159; N_Maintenance_ = 138; N_No Contact_ = 129; Number of observations used in analyses = 422.

Abbreviations: CI, confidence interval; IFG, impaired fasting glucose; IGT, impaired glucose tolerance; M, month; OR, odds ratio; SCT, Social Cognitive Theory; STD, standard.

^a^ Population-averaged Generalized Estimation Model.

^b^ Within-Person change from Baseline to 15 Months.

* p<0.05

** p<0.01.

After three months of RT, approximately 34% of the study sample achieved normoglycemia. This prevalence of normoglycemia was maintained through the Maintenance (32%) and No-Contact phases (30%). Although, the SCT group had slightly higher odds of achieving normoglycemia, there were no significant differences between groups (**Table 3**). Participants with isolated IFG or IGT had greater likelihood of achieving normoglycemia than those with combined IFG and IGT.

Both groups demonstrated significant improvements in muscular strength (p<0.001), but there were no group differences in strength change (Table 2). Strength increases were evident month 3, and maintained through month 15. These absolute increases represent improvements of 21% and 14% for chest and leg press, respectively. There were no significant group differences in body weight change over time. Body fat (%) decreased, and fat-free mass (%) increased, in both groups from baseline to month 3, which was maintained in the Standard group though month 15. Waist circumference decreased in both groups baseline to month 15. However, there were no group differences in these outcomes. Among trial participants, there was an 18% increase in the odds of reverting to normoglycemic for each 1% increase in fat-free mass (Table 3).

No group differences were noted in blood pressure, self-reported physical activity level or dietary intake, other than in percentage of energy from protein (p = 0.05). However, increases in reported physical activity (including RT) were noted in both groups from baseline to month 3, and from baseline to month 15.

Four serious adverse events were reported during the 15-month trial, which did not differ by group (**Table 4**). Three of these involved musculoskeletal and/or joint pain persisting for more than 3–4 days.

**Table 4:** Adverse Events.

	SCT group (n = 79)	Standard group (n = 80)	No group (n = 11)
Serious adverse events*	2(1)	2(1)	0(0)
Heart attack-related symptoms–Chest pain, difficulty breathing, fatigue	0	1	0
Musculoskeletal pain & difficulty breathing	0	1	0
Prolonged musculoskeletal pain	1	0	0
Prolonged joint pain	1	0	0
**Injury or musculoskeletal discomfort**	26(15)	26(15)	2(1)
Side effects and complaints			
Shoulder pain	3(2)	3(2)	0(0)
Aggravation of preexisting arthritis	2(1)	2(1)	0(0)
Tendonitis	1(1)	0(0)	0(0)
Back pain	6(4)	4(2)	1(1)
Ligament or tendon tear/pain	0(0)	3(2)	0(0)
Pinched nerve (sciatic, femoral, or cervical)	2(1)	1(1)	0(0)
Musculoskeletal injury due to accident while exercising	0(0)	2(1)	0(0)
Musculoskeletal injury due to accident outside of RT program	0(0)	2(1)	1(1)
Inflammation/swelling	2(1)	2(1)	0(0)
Other musculoskeletal discomfort	10(6)	7(4)	0(0)
**Other medical events**	3(2)	3(2)	0(0)
Surgery (heart stent, foot, hand, melanoma)	2(1)	2(1)	0(0)
Heart attack-related symptoms	0(0)	1(0.6)†	0(0)
Other	1(0.6)	0(0)	0(0)

Notes: Data are number (percentage) of participants. The “No group” category indicates participants who withdrew from study during the Initiation phase, before randomization occurred. RT = resistance training.

The difference in adverse events between Standard vs. SCT was not significant

[χ^*2*^ (*df* = 2, *N* = 159) = 0.44 (*p* = 0.80)].

* Serious unanticipated or anticipated problems, including study-related prolonged (>3–4 days) muscle pain.

† Reported after check-up with primary care physician; all tests were normal.

## Discussion

The findings of this randomized, controlled trial suggest that after adopting RT, twice per week using a 12-set, whole body protocol, the SCT-based approach was not more effective for maintenance in community-based settings. Overall, a significant reduction in prediabetes prevalence was demonstrated among previously sedentary overweight and obese older adults with prediabetes. Significant improvements in muscular strength were demonstrated, and maintained throughout the 15-month trial period. Importantly, about one-third of participants were normoglycemic at month 15. Improvements in other health outcomes, such as body composition and physical activity level, were also noted.

Contrary to our hypothesis, the SCT-based approach did not demonstrate greater effectiveness than the lower-dose standard care maintenance intervention. Although a formal cost effectiveness analysis was not performed, program delivery costs were estimated to be $1200 per participant for the initial 3-month supervised RT initiation period, $595 per participant for the SCT transition and maintenance condition, and $160 per participant for the Standard transition and maintenance condition [22]. The lower cost suggests this approach could be extended in future applications with long-term maintenance of unsupervised training noted as a critical research and translation area for prevention and treatment of chronic diseases and disorders [35].

Intensive combined diet and physical activity promotion programs are recommended to reduce type 2 diabetes [4]. Our findings suggest that RT alone may represent an effective single-component strategy to reduce prediabetes prevalence and thus, type 2 diabetes risk. The Diabetes Prevention Program (DPP) lifestyle intervention resulted in a prevalence of normoglycemia of about 40% in years 1 and 2 [36]. In contrast to our findings, the DPP lifestyle and metformin interventions reduced fasting glucose concentrations [36], which may suggest that a variety of diabetes prevention strategies are needed depending upon prediabetes phenotype [21], program accessibility, resources, and individual preferences.

Strength declines markedly with advancing age; the clinical implications of poor muscular strength include impaired mobility and limitations in activities of daily living, such as rising from a chair [37], and increased risk of type 2 diabetes [35]. Only 24% of US adults over age 45 report engaging in muscle strengthening activity two or more times per week [38], and less than 5% of adults over age 45 report meeting muscle strengthening recommendations of twice per week which includes all seven major muscle groups [39]. Yet, older adults who meet twice/week RT guidelines have a 46% lower odds of all-cause mortality than those who do not [12], and improved physical functioning [13].

Strengths of this trial include the high retention rate, a low rate of serious adverse events across about 12,500 training sessions, a theoretically-based approach, and the efficacy/effectiveness design, which may facilitate translation into clinic- and community-based interventions. Limitations of this investigation include a predominantly white, female, well-educated sample. The trial also did not include a control group, who received no intervention over the 15-month trial period. Findings cannot be generalized to younger, less-educated populations or to minorities, who may be at greater risk for developing type 2 diabetes. It is also unknown if the reduced prevalence of prediabetes was maintained beyond the 15-month trial period, however, findings from the Diabetes Outcomes Prevention Program indicate that even transient improvements in blood glucose homeostasis are associated with reduced risk of future diabetes [40].

## Conclusions

It has been estimated that 70% of individuals with prediabetes will progress to type 2 diabetes [1]. Prediabetes affects more than half of adults over 65 years of age [2], thus effective strategies to reduce type 2 diabetes risk among older adults with prediabetes are needed. The results of several 10-year cost effectiveness analyses from the DPP concluded that lifestyle interventions to reduce diabetes risk among high-risk adults are cost effective [41, 42]. Potential challenges in implementing multi-component interventions, such as the DPP, include program costs, resource limitations, and accessibility. Lower costs are possible with group-based programs and with clinic- and community-based programs [43]. Our findings suggest that the adoption and maintenance of RT, using a higher-contact SCT-based maintenance approach, is not more effective in reducing prediabetes prevalence than a lower-contact approach, with overall results comparable to that noted in the lifestyle intervention group of the DPP [36], as well as for improving muscular strength and body composition among older, overweight, prediabetic adults.

## Supporting information

S1 File Institutional Review Board Research Protocol. (DOC)

S2 File Competing Interest Statement. (DOCX)

S3 File CONSORT 2010 Checklist. (DOC)

S4 File Published Study Protocol. (PDF)
