# Supplementary material for: Resist diabetes: A randomized clinical trial for resistance training maintenance in adults with prediabetes
Source: PLoS One. 2017 Feb 23;12(2):e0172610. doi: 10.1371/journal.pone.0172610 (PMC5322950; doi:10.1371/journal.pone.0172610)
Supplement: S2 File — (DOCX) [file pone.0172610.s002.docx]

Competing Interests: One of the authors (SGW) is affiliated with PCR, Inc. This does not alter our adherence to all PLOS One policies on sharing data and materials.
