# Supplementary material for: Resist diabetes: A randomized clinical trial for resistance training maintenance in adults with prediabetes
Source: PLoS One. 2017 Feb 23;12(2):e0172610. doi: 10.1371/journal.pone.0172610 (PMC5322950; doi:10.1371/journal.pone.0172610)
Supplement: S4 File — (PDF) [file pone.0172610.s004.pdf]

Provided for non-commercial research and education use.
Not for reproduction, distribution or commercial use.

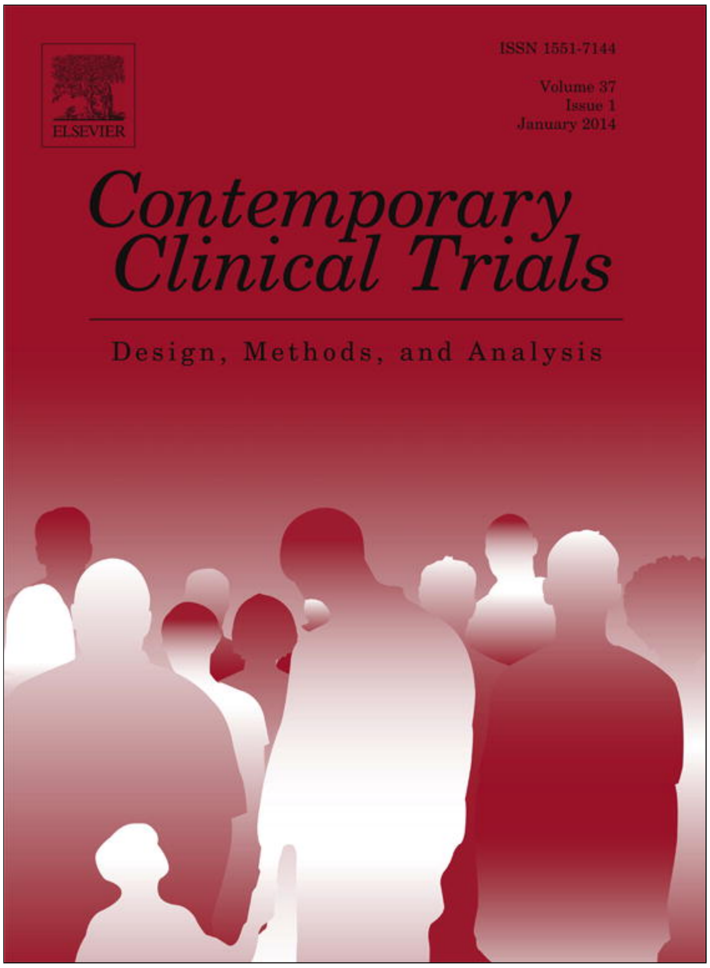

This article appeared in a journal published by Elsevier. The attached copy is furnished to the author for internal non-commercial research and education use, including for instruction at the authors institution and sharing with colleagues.

Other uses, including reproduction and distribution, or selling or licensing copies, or posting to personal, institutional or third party websites are prohibited.

In most cases authors are permitted to post their version of the article (e.g. in Word or Tex form) to their personal website or institutional repository. Authors requiring further information regarding Elsevier's archiving and manuscript policies are encouraged to visit:

http://www.elsevier.com/authorsrights

Author's personal copy

Contemporary Clinical Trials 37 (2014) 19–32

Contents lists available at ScienceDirect

Contemporary Clinical Trials

journal homepage: www.elsevier.com/locate/conclintrial

The Resist Diabetes trial: Rationale, design, and methods of a hybrid efficacy/effectiveness intervention trial for resistance training maintenance to improve glucose homeostasis in older prediabetic adults

Elaina L. Marinik [a,*], Sarah Kelleher [b], Jyoti Savla [c], Richard A. Winett [b], Brenda M. Davy [a]

[a] Department of Human Nutrition, Foods and Exercise, Virginia Tech, Blacksburg, VA 24061, United States
[b] Department of Psychology, Virginia Tech, Blacksburg, VA 24061, United States
[c] Department of Human Development, Virginia Tech, Blacksburg, VA 24061, United States

ARTICLE INFO

Article history:
Received 29 July 2013
Received revised form 7 November 2013
Accepted 9 November 2013
Available online 16 November 2013

Keywords:
Diabetes
Glucose metabolism
Strength
Body composition
Self-regulation
Disease risk reduction

ABSTRACT

Advancing age is associated with reduced levels of physical activity, increased body weight and fat, decreased lean body mass, and a high prevalence of type 2 diabetes (T2D). Resistance training (RT) increases muscle strength and lean body mass, and reduces risk of T2D among older adults. The Resist Diabetes trial will determine if a social cognitive theory (SCT)-based intervention improves RT maintenance in older, prediabetic adults, using a hybrid efficacy/effectiveness approach. Sedentary, overweight/obese (BMI: 25–39.9 kg/m^2) adults aged 50–69 (N = 170) with prediabetes (impaired fasting glucose and/or impaired glucose tolerance) completed a supervised 3-month RT (2×/wk) initiation phase and were then randomly assigned (N = 159; 94% retention) to one of two 6-month maintenance conditions: SCT or standard care. The SCT intervention consisted of faded contacts compared to standard care. Participants continue RT at an approved, self-selected community facility during maintenance. A subsequent 6-month period involves no contact for both conditions. Assessments occur at baseline and months 3 (post-initiation), 9 (post-intervention), and 15 (six months after no contact). Primary outcomes are prediabetes indices (i.e., impaired fasting and 2-hour glucose concentration) and strength. Secondary measures include insulin sensitivity, beta-cell responsiveness, and disposition index (oral glucose and C-peptide minimal model); adherence; body composition; and SCT measures. Resist Diabetes is the first trial to examine the effectiveness of a high fidelity SCT-based intervention for maintaining RT in older adults with prediabetes to improve glucose homeostasis. Successful application of SCT constructs for RT maintenance may support translation of our RT program for diabetes prevention into community settings.

© 2013 Elsevier Inc. All rights reserved.

1. Introduction

Diabetes prevalence is increasing at an alarming rate. Older individuals (>65 years) account for the largest segment of the population with type 2 diabetes (T2D), with a prevalence of ~27% [1]. Prediabetes, defined as impaired fasting glucose (IFG) or impaired glucose tolerance (IGT), affects ~79 million older, overweight adults [1]. Furthermore, up to 70% of individuals with prediabetes may advance to T2D [2]. Thus, interventions to help prevent or reduce the occurrence of T2D are urgently needed.

Past reviews of the health benefits of resistance training (RT) indicate that it is a prime intervention for older adults [3]. Brief, whole-body RT protocols consistent with current American

* Corresponding author at: 221 Wallace Hall, 0430, 295 West Campus Drive, Blacksburg, VA 24061. Tel.: +1 540 553 5578.
E-mail address: emarinik@vt.edu (E.L. Marinik).

1551-7144/$ – see front matter © 2013 Elsevier Inc. All rights reserved.
http://dx.doi.org/10.1016/j.cct.2013.11.006

Author's personal copy

College of Sports Medicine (ACSM) guidelines [4] could increase strength and muscle mass and reduce the risk of sarcopenia, insulin resistance, and loss of bone mineral density [5–7]. RT is increasingly recognized as an important treatment component for T2D [8] to improve glycemic control and insulin sensitivity via increased muscle cross-sectional area [9], increased lean body mass [10,11] and improvements in muscle metabolic properties [12]. RT is particularly beneficial for older, prediabetic adults given the loss of lean body mass and worsening of glucose tolerance with aging. Beneficial effects of RT have been noted without changes in body weight/fatness and cardiorespiratory fitness [9,10,13–15]. Since both reductions in lean body mass and worsening of glucose tolerance are commonly observed with advancing age [16,17], RT regimens that safely and efficiently maintain or increase lean body mass may prevent or delay the development of impaired glucose homeostasis [18].

RT can be effectively initiated in well-supervised settings, yet few theory-based studies exist which demonstrate effective maintenance of RT in minimally supervised settings [19]. While social cognitive theory (SCT) has been frequently applied to physical activity (PA) and to some extent aerobic training, it has been minimally applied to RT. Unique aspects of RT such as its precision and intensity, and time and place specificity may require greater and differential use of self-regulation skills in both the short-term and long-term than aerobic training. Different constructs and variables also may have differential importance and impact in initiating and then maintaining RT.

Current diabetes prevention programs have had difficulty translating and continuing the beneficial lifestyle outcomes evident in a supervised clinical setting to largely unsupervised community settings [20–27]. The Resist Diabetes trial attempts to demonstrate that the RT protocol used in a supervised clinical setting can be successfully translated and maintained in community settings by implementing an SCT-based maintenance intervention. The intervention is one of few studies [8] to apply familiar SCT constructs, such as self-efficacy, outcome expectancy (OE), self-regulation, and behavioral constructs, to the initiation and maintenance of RT in older adults. The Resist Diabetes trial will evaluate a 15-month SCT-based intervention for the maintenance of RT in older overweight adults with prediabetes, using a hybrid efficacy/effectiveness approach.

2. Aims and hypotheses

The overall aim of this phase II clinical trial is to demonstrate the efficacy and effectiveness of a high fidelity SCT-based intervention for initiating and maintaining RT in older adults with prediabetes to improve glucose homeostasis. It is hypothesized that SCT-based follow-up with faded contact will produce better outcomes than the Standard Care follow-up at 9-month and 15-month assessments. It is also hypothesized that improvements in glucose homeostasis and strength from RT will be mediated by adherence, self-efficacy, and use of self-regulation strategies.

Primary outcome measures are indices of prediabetes (i.e., impaired fasting and 2-hour blood glucose concentration) and strength. Secondary measures include insulin sensitivity, β-cell responsiveness, and disposition index as determined by the oral glucose and C-peptide minimal model; RT adherence; fat-free mass; lean body mass gain; other indicators of health and metabolic fitness; and SCT measures.

3. Study design

3.1. Overview

The Resist Diabetes study is a 15-month randomized controlled trial including men and women aged 50–69 years (N = 170) with prediabetes, defined as exhibiting either IFG (fasting glucose = 95-125 mg/dl) and/or IGT (2-hour glucose = 140–199 mg/dl), and who met all other inclusion criteria. All participants first followed the same standard, supervised 3-month initiation phase with RT (detailed in Section 3.6.2 RT protocol — initiation phase). After the 3-month initiation phase, participants (N = 159; 94% retention) were randomly assigned to one of two maintenance conditions for 6 months: 1. a long-term, higher fidelity, SCT intervention using interactive, self-regulation procedures (e.g., goal setting, monitoring, reporting feedback, planning, problem solving) with tailored web-based and faded personal contact, or 2. A more standard, usual care condition with SCT content (e.g., didactic instruction in problem solving), generic web-based and more minimal contact. Thus, the two conditions shared SCT content but differed on the degree of interactivity, tailoring, and dose of contact. A 4-week transition phase following randomization prepared participants to continue the RT protocol at a self-selected but project-approved community/public health facility. For both conditions, contact ended after 6 months, but the participants are expected to continue RT at their respective facilities. The assessments are completed at baseline, at the end of the common initiation phase (3 months), at the end of the different maintenance intervention phase (9 months), and six months after all contact has ended (15 months from baseline), providing an assessment of maintenance with differential faded contact and with no further contact. If group differences are detected in the primary outcomes, we acknowledge that it will be uncertain if we can attribute these differences to the high fidelity SCT intervention or the degree of contact received by each group.

The study design, recruitment, and randomization overview is presented in Fig. 1. Of those responding to study advertisements, accessing our informational website, and establishing online accounts (N = 1046), 846 individuals initiated the preliminary online eligibility screening process. Of those, 561 individuals were disqualified during the online screening process and 285 individuals consented to participate and were screened at a baseline assessment clinic. One hundred seventy individuals met prediabetes eligibility criteria [28] and enrolled in the supervised initiation phase. After the 3-month initiation phase, 159 participants were randomized to one of the two conditions (SCT: N = 79; Standard: N = 80). Ten participants dropped out of the initiation phase for various reasons (Fig. 1). At present, up to the 9-month assessment clinic, overall retention is 81%.

3.2. Recruitment and screening

Adults aged 50–69 years were recruited from Roanoke, Virginia and its surrounding communities (Table 1). Recruitment began in January 2011 and ended September 2012.

Author's personal copy

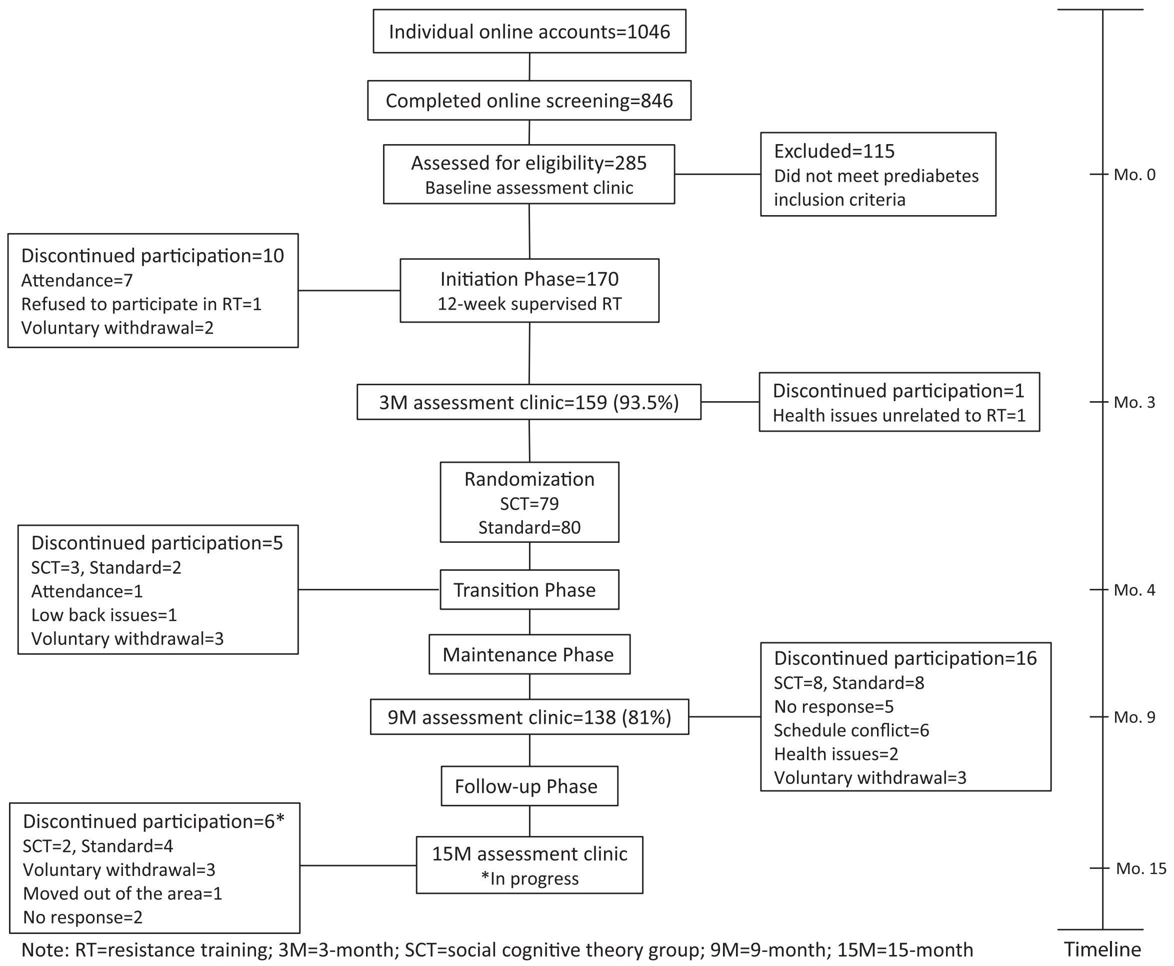

Fig. 1. Resist diabetes study consort diagram.

Recruitment methods included newspaper advertisements, advertising at workplaces and community churches, and direct mail in targeted zip codes (within a 15-minute drive from the research facility) to households with non-smokers and at least one adult within the required age range. Recruitment took place in four waves, with each recruitment period lasting about four months. Recruitment informational materials directed potential participants to a study informational website, which allowed individuals to determine if they met eligibility requirements via an online self-screening process. Prior to accessing the online screening questions, potential participants completed a brief online informed consent process. If they remained interested in participating after reading this information, individuals were invited to create a personal account on the website and complete a comprehensive online screening questionnaire which inquired about demographics (i.e., age, gender, ethnicity, level of education, income and occupation), height and weight, usual PA level (including RT), cardiovascular disease (CVD) symptoms, health and medical history, medication usage, and the AHA/ACSM Health/Fitness Facility preparticipation screening questionnaire [29]. To be eligible, individuals were required to have internet access for screening and participation, as some intervention components were delivered and completed online.

3.3. Eligibility

Study inclusion and exclusion criteria are listed in Table 2. Eligible individuals were apparently healthy (no known presence of heart disease), with no symptoms of CVD (chest discomfort, dizziness, shortness of breath, leg discomfort consistent with claudication) as defined by the American Heart Association [30]. Individuals were excluded if they indicated that they were current smokers, had been diagnosed with CVD, diabetes, pulmonary, liver or kidney disease, or other life-threatening illnesses or conditions during the online screening process. Additional exclusion criteria included conditions restricting PA or the ability to RT, such as orthopedic injuries or musculoskeletal disabilities, and taking any medications known to influence energy metabolism (e.g., beta blockers) or body weight or composition (e.g., thyroid replacement, antidepressants). Individuals taking other commonly prescribed medications, such as those used to treat dyslipidemia or hormone replacement therapy, were eligible for participation provided that they had been on a stable dose of the medication for an extended period of time (e.g., >1 year). Individuals with hypertension whose blood pressure (BP) was adequately controlled (i.e., <140/<90 mm Hg) with antihypertensive medications were permitted to participate. Individuals who appeared eligible following the online screening were

Author's personal copy

Table 1
Resist diabetes: participant characteristics at baseline (N = 170)[a].

Age (years)	59.5 ± 5.4
Gender	
Female	124 (73%)
Male	46 (27%)
Weight (kg)	93.3 ± 13.9
BMI (kg/m^2)	33.0 ± 3.8
Systolic BP (mm Hg)	130.9 ± 15.0
Diastolic BP (mm Hg)	75.6 ± 8.9
Fat percent (%)	43.8 ± 6.9
Fat mass (kg)	40.6 ± 8.4
Fat-free percent (%)	56.2 ± 6.9
Fat-free mass (kg)	52.1 ± 10.4
WC (cm)	109.1 ± 10.3
Chest press 3RM (lb)	74.2 ± 25.5
Leg press 3RM (lb)	310.8 ± 79.1
Fasting glucose (mg/dL)	101.7 ± 8.3
2 h glucose (mg/dL)	142.3 ± 36.3
Prediabetes criteria	
IFG	82 (48.2%)
IGT	21 (12.4%)
Both IFG & IGT	67 (39.4%)
Race	
White	159 (93.5%)
Black	10 (5.9%)
Other	1 (0.6%)
Ethnicity	
Hispanic	2 (1%)
Non-Hispanic	168 (99%)
Education level	
High school	6 (4%)
College (partial or completed)	111 (65%)
Grad/professional degree	53 (31%)

Note: BMI = body mass index; BP = blood pressure; WC = waist circumference; 3RM = 3 repetition maximum; IFG = impaired fasting glucose; IGT = impaired glucose tolerance.
[a] Continuous variables expressed as means ± SD and categorical variables are expressed as frequency (%).

required to obtain the written consent of their personal care physician (PCP) using a standardized medical clearance form prior to enrollment, which also described the nature of the study. Additional eligibility criteria (i.e., prediabetes status) were assessed during the initial laboratory assessment session.

Table 2
Resist diabetes: participant eligibility criteria.

Inclusion criteria:
- Men and women, 50–69 years of age
- Sedentary: Moderate PA < 60 min 2 d/wk; Vigorous PA < 30 min 2 d/wk
- BMI: 25 kg/m^2 ≥ BMI < 40 kg/m^2
- Prediabetes: IFG (Fasting = 95–125 mg/dl) and/or IGT (2-hr = 140–199 mg/dl)
- No RT in the past 12 months
- Internet access

Exclusion criteria:
- Current smokers
- Current diabetes diagnosis or use of diabetes medications (e.g., metformin, insulin)
- History of CVD
- Active cancer or treatment of cancer
- Short-term (<1 year) use of medications affecting weight or glucose metabolism (e.g., thyroid replacement, hormone replacement, anti-depressants)
- Orthopedic or musculoskeletal contraindications to participate in RT
- Uncontrolled HTN (BP > 160/100 mm Hg)
- Pregnancy

Note: IFG = impaired fasting glucose; IGT = impaired glucose tolerance; CVD = cardiovascular disease; RT = resistance training; HTN = hypertension; BP = blood pressure.

3.4. Assessment clinics

3.4.1. Testing sessions

Individuals eligible following the online screening who submitted a signed medical clearance form to the project director (PD) were scheduled for a baseline assessment clinic at Virginia Tech (VT) Riverside in Roanoke, VA. The assessment clinic consisted of two testing sessions. Potential participants received a reminder about their upcoming assessment clinic and were provided preparation instructions via email. Individuals were asked to arrive in a fasted state (no food or caloric beverages for 12 h prior to appointment), consume 2–3 cups of water the evening before and morning of their appointment, and abstain from exercise and alcohol 48 and 24 h, respectively, prior to their appointment. The first visit included a detailed health and medical history and assessments of height, weight, body mass index (BMI), and BP, as well as fasting glucose and glucose tolerance during a 2-hr oral glucose tolerance test (OGTT) to confirm eligibility. To remain eligible, individuals met one or both criteria for prediabetes: 95–125 mg/dl fasting plasma glucose (FPG) concentration (based on Diabetes Prevention Program criteria; [28]) and 140–199 mg/dl 2-hr plasma glucose concentration post 75 g glucose load. Over the four waves of recruitment, 60% of participants who completed baseline laboratory testing qualified for the study. Individuals who were not eligible based on their measured BMI or plasma glucose concentrations were disqualified and did not continue to the second day of testing. Eligible individuals were then scheduled by phone for the second day of testing sessions, which included waist circumference (WC), a body composition scan, and strength tests (3-repetition maximum [RM]) at the same facility. Participants who complete any of the 2-day assessment clinics are compensated (e.g., gift cards or cash) each time. Assessments completed at baseline and at each subsequent testing session are listed in Table 3.

3.4.2. Timeline

Assessment clinics are completed by each participant at baseline and each subsequent follow-up assessment point (i.e., 3 months, 9 months, 15 months), at least 24 h after their last RT session. The 2-day assessment clinics are conducted within an average two- to ten-day period at each assessment point. Up to 5 participants are scheduled between 7–8 a.m. in 15-minute increments for day 1 testing. All OGTTs begin between 7 and 9 a.m., which is held consistent for each individual. Each participant is scheduled individually between 7 a.m. and 7 p.m. with one of the research assistants/ACSM-certified personal trainers for day 2 testing. Testing sessions for each participant last approximately 3 h on day 1 and 1–1.5 h on day 2.

3.5. Measures

The following variables are assessed at each 2-day assessment clinic, as listed in Table 3.

Author's personal copy

Table 3
Resist diabetes: assessment clinic timeline.

	Data collection time point			
	Baseline	3 months	9 months	15 month
Participant rights/information				
Informed consent document	X			
Medical clearance form	X			
Health history questionnaire	X	X	X	X
Anthropometrics				
Height	X			
Weight	X	X	X	X
Waist circumference	X	X	X	X
Body composition (DXA)	X	X	X	X
Muscular strength				
3RM chest press	X	X	X	X
3RM leg press	X	X	X	X
Blood chemistry/pressure				
Fasting glucose sample	X	X	X	X
2-h glucose sample	X	X	X	X
Blood pressure	X	X	X	X
Dietary intake				
24-hour recalls	X	X	X	X
Questionnaires				
RT health beliefs	X	X	X	X
PAQ	X	X	X	X
Trainer ratings		X		
Timeline follow-back calendar			X	X

Note: DXA = Dual-energy X-ray absorptiometry; 3RM = 3 repetition maximum; RT = resistance training; PAQ = physical activity questionnaire.

3.5.1. Anthropometrics

Height is measured without shoes to the nearest 0.1 cm using a wall-mounted stadiometer. Body mass is measured to the nearest 0.1 kg using a digital scale (Healthometer ProPlus ™, Pelstar, McCook, IL). BMI is calculated as weight (kg)/ height (m)^2. WC is measured to the nearest 0.25 cm using a Gulick tape measure at the level of the umbilicus and the mean of two measurements within 1.0 cm is recorded. Body fat percent, absolute fat mass and fat-free mass is assessed using dual-energy X-ray absorptiometry (DXA; GE Lunar Prodigy, software version 11.40.004, Madison, WI). The participants wear a hospital gown during DXA scans to standardized clothing. Premenopausal women (without a hysterectomy or complete oophorectomy) complete a urinary pregnancy test prior to each scan.

3.5.2. Blood pressure (BP)

BP measurements are conducted according to standardized guidelines [31]. Briefly, individuals are fitted with an appropriate size cuff and sit quietly for 5 min in a clinical exam room. BP measures are then obtained every 3 min using a Dinamap automated sphygmomanometer (ProCare, Model 9300, GE HealthCare, Milwaukee, WI). BP measurements continue until the mean of 2 consecutive readings is ±10 mm Hg for systolic and diastolic BP [32,33].

3.5.3. Oral glucose tolerance test & blood chemistry

Consistent with American Diabetes Association criteria [34], prediabetes is determined using FPG concentrations and 2-hour OGTT results. The fasting and 2-hour glucose concentrations are primary outcome measures. Participants are seated for the 2-hour test period. Our research nurse inserts a 20-gauge catheter with a 3-way stopcock for blood sampling into the antecubital vein. A baseline fasting glucose sample (two 7-mL plasma and two 5-mL serum vacutainers) is initially obtained (min 0). The participants then consume an 8-ounce 75 g orange-flavored glucose beverage (Fisherbrand, Fisher Scientific, Hanover Park, IL) within the first 5 min of the test. Additional blood samples are obtained at minute 10, 20, 30, 60, 90 and 120 after consumption of the glucose load according to the 7-sample OGTT [35]. A saline flush maintains patency after each blood draw. When catheter placement is not successful, a butterfly needle is used only at time 0 and 120 for blood collection. All blood collections are performed in aseptic conditions. Plasma samples are immediately placed on ice and serum samples remain at room temperature for at least 15 min after each collection timepoint.

Collected blood samples are processed on-site by a trained research technician. Blood samples are separated by centrifugation (Model 5702R, Eppendorf, Hauppauge, NY) at 2000 g for 15 min. Glucose concentrations (mg/dl) are immediately analyzed in duplicate on a YSI 2700 Select glucose analyzer (YSI Life Sciences, Yellow Springs, OH). The plasma and serum samples (4 mL each) at each timepoint are transferred to 2-mL cryogenic vials and stored at −80 °C (Revco Ultima II, Thermo Scientific, Hanover Park, IL) for later analysis of insulin and C-peptide (see Section 3.5.4 Oral glucose and C-peptide minimal model). Batch analysis of the stored plasma and serum samples will occur in the Human Nutrition, Foods and Exercise departmental Metabolic Phenotyping Core laboratory at VT after data collection is complete. Commercially-available assay kits for insulin and C-peptide will be used (Human insulin and C-peptide ELISA, ALPCO; Insulin and C-peptide IMMULITE kits, Siemens).

3.5.4. Oral glucose and C-peptide minimal model

Changes in glucose and insulin metabolism will be assessed by determination of insulin sensitivity, beta-cell responsivity, and disposition index during the OGTT at each assessment point. Insulin sensitivity (SI) will be estimated from plasma glucose and insulin concentrations using the oral glucose minimal model [35–38], while indices of beta-cell responsivity (dynamic, Φd, static, Φs, and overall Φ) will be assessed from plasma glucose and C-peptide concentrations using the oral C-peptide minimal model [35,39]. In order to determine whether insulin secretion is appropriate for the degree of insulin resistance, Φd, Φs, and Φ will also be expressed in relation to insulin sensitivity through the dynamic, DId, static, DIs, and overall DI disposition indices.

3.5.5. Dietary intake assessment

Three 24-hour dietary recalls are completed to assess habitual dietary intake at each assessment point. The first recall is completed in-person by a trained research dietitian during the OGTT. Sample pictures of dinnerware, glassware, and measuring utensils are provided to aid participants in

Author's personal copy

their serving size estimations, if necessary. The remaining two dietary recalls are completed by phone over the following two weeks by the same dietitian; recall days are unannounced according to recommended dietary assessment procedures [40]. Two weekdays and one weekend day are recalled. To date, our overall completion rate for collecting three recalls at each time point is 89%. Although dietary data collection and analysis is ongoing, preliminary analyses indicate that the mean reported energy intake (EI) of participants in our sample is ~1800 kcal/d, which is similar to that reported by this age segment in nationally representative samples [41]. Additional analyses comparing reported EI to estimated daily energy requirements indicate that reported EI was within ~8% of estimated requirements in men and within ~15% of estimated requirements in women. The Nutrition Data System for Research software (NDS-R 2010, University of Minnesota, Minneapolis, MN) is used to estimate energy, macronutrient, and micronutrient content [e.g., kcal; carbohydrate, fat and protein (g and %); cholesterol (mg); fiber (g); sodium (mg); Vitamin A, C, E (%)], and other relevant dietary variables (e.g., glycemic load, added sugars, energy density).

3.5.6. Strength tests

Strength change on the chest press and leg press resistance machines is a primary outcome measure. Three-repetition maximum (3RM) strength is assessed following ACSM guidelines and procedures at each assessment point [4]. Prior to baseline assessment, all participants had an extended orientation and practice with these movements. Following orientation, the procedures consists of a general aerobic warm-up (i.e., recumbent bike or treadmill), a specific warm-up on each machine, and then the performance of five controlled (3-second concentric, 3-second eccentric) repetitions where the last repetition is judged as 'hard' (7–8) on a Borg perceived exertion scale (RPE; 1–10; [42]). Participants then rest for 3–5 min. Resistance is increased 10%–20% beyond the resistance used for completing five repetitions on each machine. Participants subsequently perform three controlled repetitions at an RPE of 'very hard' (9–10) during the last repetition with an inability to perform a fourth repetition. If this level of perceived exertion is not reached, participants rest for 1–2 min, resistance is increased 5%–10%, and the procedure repeated. The resistance used for the 3RM on the chest press and the leg press (max. of 495 lb) is the strength measure at all assessment points. In order to assure uniformity, strength testing follows the same procedures at each assessment point.

3.5.7. Physical activity questionnaire and health beliefs survey

The participants are instructed to complete two online surveys regarding PA and their health beliefs related to RT prior to each scheduled assessment clinic. The physical activity questionnaire consists of questions on type, frequency and intensity of PA that participants may have participated in within the last 3 months [43]. The RT health beliefs survey, adapted and further developed specifically for this trial [44], rates measures of social support, self-efficacy, outcome expectations and self-regulation during periods of RT.

3.5.8. Trainer ratings

During the 3-month assessment clinic, our research assistant/personal trainers were evaluated by each participant on their conduct during the initiation phase. A 7-item trainer rating tool was used to determine scores related to competence, professionalism, helpfulness, and program satisfaction. All trainers received high mean rating scores (>6/7 point rating) throughout the study period. Trainer ratings were used to insure consistent supervision and feedback of each RT session and to determine the effect of the trainers' RT knowledge and behavior on participants' motivation to RT.

3.5.9. Feedback reports

The participants receive a 3-page feedback report in-person or by mail after the completion of each 2-day assessment clinic. The report includes a description of each measurement, categorical charts of normative or ideal results (if available), and their personal outcomes for each completed assessment measure. They also receive a summary report of their dietary intake compared to the dietary reference intakes [45]. A pamphlet containing standard nutrition information from the 2010 Dietary Guidelines for Americans [46] is also included in the feedback packet.

3.6. Resistance training protocol

3.6.1. Performance site and personal trainer supervision

All training during the supervised initiation phase was conducted in the lab–gym at VT's Riverside research facility in Roanoke, VA. The lab–gym is a 1500 sq ft training area which is equipped with aerobic exercise equipment (treadmills, recumbent bikes) and 12 Nautilus Nitro Plus resistance training machines. Research assistants/personal trainers (1 full-time and 2 part-time) were available from 7 a.m. to 7 p.m. every weekday. This allowed for 2–4 participants to be scheduled at any time, with each trainer attending to 1–2 participants during each RT session.

All the trainers were ACSM-certified and had CPR and AED training. Their primary role during the initiation phase (see Section 3.6.2 RT protocol — initiation phase) was that of a conventional trainer. The trainers were instructed on the delivery of the RT protocol and other procedures used in each session. They were also provided a trainer's manual, emphasizing the conduct of each RT session, data entry and scheduling RT sessions. Prior to supervising the study participants, the research assistants/personal trainers were observed over a 2-week period by the principal investigators (PIs) and PD during training sessions with volunteer "practice subjects". The practice sessions included exposure to each piece of Nautilus equipment used in the intervention so that the trainers became familiar with their use and proper techniques. During the initiation phase, the primary role of the trainers during a session was to provide encouragement and corrective feedback on each participant's form and the degree of apparent effort.

3.6.2. RT protocol — initiation phase

All the participants were involved in RT sessions 2 times per week for 3 months supervised by our research assistants/personal trainers in our lab–gym. The initiation phase used a whole-body RT protocol that conformed to ACSM guidelines [4] on nonconsecutive days. The training schedule was set at

Author's personal copy

the same time on Monday and Thursday or Tuesday and Friday (with Wednesday as a make-up day), allowing for at least one day of rest between RT sessions. The exercise pieces in the protocol were: leg press, leg extension, seated leg curl, calf raise, chest press, lat pulldown, row, shoulder press, seated dip, lower back, abdominal crunch and rotary torso. The protocol took approximately 35–45 min to complete. The participants were required to attend at least 17 of the 24 scheduled RT sessions (70% minimum adherence) to participate in the 3-month assessment clinic and progress to the next phase of the study.

The first two sessions (week 1) of the initiation phase involved mastery experiences for RT-trainer modeling, guided practice, corrective feedback on participant's repetition, duration, and range of motion, overall form, and practice making a good-to-high degree of effort on exercises using the RPE scale as a basis. The participants also received both verbal instructions and a printed manual describing the principles of RT within the 'intrinsic' (i.e., high degree of effort and fatigue within the targeted muscle group) training model. This approach follows from recent research showing that the stimulus for muscle protein synthesis and hypertrophy appears to be related to a high degree of effort and fatigue and not a specific RM as assumed within the traditional, 'extrinsic' (i.e., predetermined percent of RM and/or repetitions) weightlifting model [47]. The resistance for each machine during these sessions was set by the trainers at ~50% of the starting resistance determined during the baseline assessment clinic (see Section 3.5.6 Strength tests). Thereafter, all the sessions were supervised and all the records of each participant's training session were entered into an online database. In week 2, training began with resistance established for each exercise for each participant during assessment. Subsequently, the participants could only increase resistance 5% on a given exercise after completing at least eight 3-second concentric, 3-second eccentric contractions on the exercise (with good form) for 2 consecutive sessions or 2 of 3 RT sessions with a high RPE. Overall, the participants were expected and judged to perform all 12 exercises at moderate resistance for 8–12 repetitions with good form and a high degree of effort to concentric failure during each RT session over the 3-month Initiation phase.

3.6.3. Rationale for the selection of exercise dose/volume

Compelling evidence shows that relatively simple, brief, lower volume RT protocols performed 2–3 times per week and focused on compound movements that affect multiple muscle groups can provide an adequate stimulus to promote strength and hypertrophy [48]. This 'intrinsic', effort-based RT approach, itself a paradigm shift from the 'extrinsic' weightlifting model, focuses on proper form and degree of effort. In practice, middle aged to older adults can, in this manner, safely RT 2–3×/wk with whole-body routines taking about 35–45 min. In order to assure sufficient volume, our RT protocol described above (Section 3.6.2 RT protocol — initiation phase) includes multiple exercises for major muscle groups for one set each. Note that traditional RT protocols are described as training at a specific RM, such as 80% of 1RM in a given exercise. As pointed out in the ACSM guidelines [4], however, this approach has its limitations. Because of a number of factors, different people will be able to perform a different number of repetitions with a given RM. There also are intra-individual differences so that for different exercises at the same RM, a different number of repetitions may be performed. Therefore, we believe we have delivered a high quality, standard RT initiation program for our older, sedentary prediabetic participants.

3.7. Participant safety

3.7.1. IRB approvals and confidentiality

All the study procedures were approved by the Institutional Review Board (IRB) at Virginia Polytechnic Institute and State University. Potential participants provided informed consent three times prior to enrolling in the Resist Diabetes study. Interested individuals were required to read an online version of the informed consent document and acknowledge consent prior to entering the online screening process. As previously described (see Section 3.3 Eligibility), potential participants who remained eligible after completing the online screening questionnaire also obtained medical clearance from their PCP. The standardized form described the study rationale, purpose and protocol, and was used to obtain the physician's signature of clearance for participation and contact information. These individuals also provided written consent prior to completing day 1 of the baseline assessment clinic. If an individual remained eligible after the baseline assessment clinic, verbal consent was obtained to continue study procedures.

All self-reported and online assessments were treated as confidential. Participant folders containing medical history and collected data, and all consent forms and medical clearance forms were stored separately in secured, locked filing cabinets accessible only to project personnel. Electronic copies of data were password protected, filed with no identifying information, and stored on computers with firewall protection. No participant social security numbers were collected at any point of the study.

3.7.2. Adverse events and data and safety monitoring protocols

In the event of an adverse event (AE; e.g., cardiovascular event, prolonged musculoskeletal soreness or pain), participants were instructed to immediately contact a member of the study staff (e.g., personal trainer, PD, Medical Director) by phone or email. For emergencies, participants were instructed to go directly to the emergency room or call 911. During the initiation phase, research assistants/personal trainers were asked to record and report any signs of minor or major problems during RT to the PD and/or PIs. For "minor" prolonged problems (i.e., prolonged muscle soreness), we responded immediately and recommended that participants stop the RT program and consult with their PCP. For any problem, we initiated a follow-up contact to assure participants were able to follow up with a health care provider. We also required medical clearance for such participants to proceed in the RT program. Records of these reports are maintained to determine if a specific participant repeatedly reports problems. All the contacts were secure, stored confidentially, monitored daily, and accessible only by the program staff. All AEs were reported to the IRB within 24 h of notification from the participant, as required by the university; thus far, only four AE reports (shortness of breath and chest

Author's personal copy

pain; persistent back pain with shortness of breath; substantial and prolonged pain and stiffness in hip and leg; prolonged shoulder pain) potentially related to RT have been submitted to the IRB. A report including descriptions of AEs is provided every six months to the trial's Data Safety and Monitoring Board (Members: Project Medical Director and two faculty not otherwise involved with the trial, with expertise in Exercise Physiology and Psychology) in order to review study progress and ensure participant safety.

4. SCT and standard conditions

The participants were randomized to either an SCT-based intervention or standard, usual care condition after completing the 3-month assessment clinic but prior to the start of the transition phase. A stratified random assignment procedure was used with sex and responsiveness (i.e., initial mean strength gains on chest press and leg press of <15%, 15% to <35%, or ≥35%) as grouping variables. Table 4 presents the characteristics (as collected at the 3-month assessment clinic) of our randomized participants by intervention group. Fasting glucose concentration assessed at 3 months was higher for females assigned to the SCT group compared to the standard care group (103.6 mg/dl vs. 99.6 mg/dl, respectively).

The following sections describe the SCT intervention and the standard condition. The transition phase lasted four weeks, comprised of nine sessions for SCT participants and three sessions for standard participants. Following transition, the maintenance phase lasted six months, followed by six months of no contact. Table 5 presents the differences between the SCT and standard intervention groups during the transition phase and also shows how each SCT element was operationalized and employed.

4.1. SCT intervention

4.1.1. A theory-based approach

Consistent with SCT, this hands-on and highly individualized intervention focuses on self-efficacy, OE, and self-regulation related to RT. Our approach to training and problem solving was guided by multiple domains of self-efficacy (i.e., task self-efficacy, barrier self-efficacy, and self-regulatory efficacy) and OE (i.e., positive instrumental/social OE, negative instrumental/social OE, and affective response expectancy). We also facilitated the use of self-regulation strategies necessary to regularly resistance train 2×/wk, including planning, goal setting, and problem solving.

4.1.2. Transition phase

The nine SCT transition phase sessions (see Table 6) included RT workouts with and without a personal trainer at both the lab–gym and newly self-selected community/public health facility. Across these sessions, the participants gradually transitioned from RT with a trainer, to training alone but with a trainer present, and lastly, to training alone with no trainer present in the new facility. This phase involved faded contact meant to ease each participant's transition from the lab–gym to his or her selected facility. The participants were responsible for paying the monthly membership fees associated with using the self-selected facility. The overall objective was to optimize self-efficacy, use strategies to change or improve ecological factors, and to make progress in RT with some potential minor modifications in the protocol to improve the likelihood of continued training.

Each transition session began with a brief review of the previous RT session, the stated goals for the current session, and relevant data entry. Each session ended with a review of the RT session; reporting of workout data, including notes on form and RPE; and collaborative development of plans and goals for the next session. In addition, SCT participants reported an overall expectancy rating (0–10) to continue to RT twice weekly after each RT session. Of note, the goal setting was individualized while generally following the guideline of increasing resistance by ~5% for an exercise when eight 3-second concentric, 3-second eccentric repetitions were completed with a given resistance for two consecutive RT sessions or 2 of 3 sessions. Tailored feedback on each participant's workouts and progress were provided via the Resist Diabetes website based on plans and meeting goals (discussed below in Section 4.1.4 RT-tracking software).

During transition, the research assistants/personal trainers engaged in problem solving with each participant, when

Table 4
Participant characteristics at randomization (month 3) by treatment group (N = 159).

	Female		Male	
	SCT<br>N = 59	Standard<br>N = 56	SCT<br>N = 21	Standard<br>N = 23
Age (years)	58.8 ± 5.5	59.0 ± 5.3	61.7 ± 5.4	61.3 ± 4.6
Weight (kg)	89.4 ± 11.7	89.7 ± 12.7	102.2 ± 13.6	103.9 ± 14.6
BMI (kg/m^2)	33.3 ± 3.9	33.1 ± 4.2	32.1 ± 3.9	32.8 ± 3.0
Fat percent (%)	46.3 ± 4.1	46.6 ± 3.9	34.0 ± 4.4	34.9 ± 4.9
Fat mass (kg)	41.3 ± 7.2	41.6 ± 8.0	35.0 ± 8.5	36.3 ± 9.0
Fat-free percent (%)	53.7 ± 4.1	53.4 ± 3.9	66.0 ± 4.4	65.1 ± 4.9
Fat-free mass (kg)	47.5 ± 6.4	47.2 ± 5.8	66.9 ± 6.3	66.4 ± 7.4
WC (cm)	106.4 ± 10.6	107.6 ± 11.0	110.6 ± 10.2	112.4 ± 9.2
Fasting glucose (mg/dL)	103.6 ± 10.9	99.6 ± 10.0†	97.9 ± 8.2	99.0 ± 7.1
2 h glucose (mg/dL)	136.2 ± 34.1	137.8 ± 36.1	123.8 ± 34.8	132.7 ± 40.3
Chest press 3RM (lb)	76.7 ± 14.8	77.8 ± 13.8	137.9 ± 23.1	139.8 ± 24.9
Leg press 3RM (lb)	330.7 ± 74.3	330.4 ± 62.7	468.2 ± 45.6	463.7 ± 40.3

Note: Values are means ± SD. BMI = body mass index; WC = waist circumference; 3RM = 3 repetition maximum.
† $p < 0.05$ between female groups using one-way ANOVA.

Author's personal copy

Table 5
Social cognitive theory elements operationalized and employed by intervention groups.

SCT constructs, program structure, relevant measure	SCT	Standard (limited contact after initiation phase)
Knowledge, information; Knowledge scale, self-efficacy	Demystifying RT. Simple principles of training and progression. Probable course of improvement and issues in maintenance. Delivered in verbal and print form.	Demystifying RT. Simple principles of training and progression. Probable course of improvement and issues in maintenance. Delivered in verbal and print form.
Self-regulation in session — subset of SR scale	Focus on form and effort; based on progress, plus repetitions and resistance; plan next session.	Focus on form and effort; based on progress, plus repetitions and resistance; plan next session.
Self-regulation outside session — subset of SR scale after supervised training ends	Problem solving to choose strategies given barriers to training. Report on strategies, provide feedback, stay with or choose new strategies.	Brief print manual about problem solving.
Self-monitoring — participants' records	Record each workout plus specific strategies chosen; notes on session to improve next session.	Generic information about the process provided plus downloadable workout forms from the website.
Goal setting, feedback — participants' goals	Receive automated tailored feedback on each session and suggested goals for each exercise.	Generic information about the processes provided.
Ecological — strategies selected and used	Initial and continued assessment to develop strategies to overcome barriers and facilitate RT.	Information included in brief print manual about problem solving.
Expectancy to continue training scale	Continuous assessment of expectancy for training 2×/wk. Use expectancy rating as a gateway to strategies provided on the website and by the follow-up coordinator.	No assessments or strategy selection.
Transfer of training — measure ratings of comfort in new facility and how well RT will fit schedule	Hands-on sessions in new facility. Attention to how the new machines work, comfort level in new facility and fitting RT into schedule.	One orientation session in the new facility and tips to continue.
Protocol — participants' modifications	Modifiable with feedback within limits to maintain or improve expectancy.	N/A
Continued contact	Faded contact with follow-up coordinator revolving around SCT constructs.	Limited faded didactic contact with the follow-up coordinator.

Note: SCT = social cognitive theory; SR = self-regulation; RT = resistance training.

necessary, based on questions or problems that arose and reported barriers to RT maintenance, including reported self-efficacy or affect and ecological factors. Discussion points may have included work and family responsibilities and schedules, time for rest and recovery, and support from other people. A strategy may have been chosen and used to correct or change circumstances or to improve circumstances that were adequate but could have been more facilitative. Based on experience with using the strategy, the strategy may have been retained or modified, or a new strategy was chosen. Thus,

Table 6
Social cognitive theory and standard group transition phase sessions.

Sessions for SCT	Procedures	Sessions for standard	Procedures
1–2	Train with trainer and receive session feedback. Review training plan and goals for the day. Together enter all training data into lab–gym computer. Enter expectancy rating. Problem solve for barriers. Enter strategies for barriers. After second session, can minimally modify basic protocol to improve expectancy. Collaboratively, plan next session. Receive feedback on workout based on plan and meeting goals by second session.	1	Meet trainer for initial didactic session at lab-gym. Receive manual on frequent RT problems and typical solutions. Receive general information about local facilities to join. Review general information available on study website.
3–4	Train alone with trainer present. Enter all training data in lab–gym alone and receive feedback based on plan and meeting goals. Plan next workout given expectancy rating and progress.		
4	Choose facility with trainer to continue to train alone after transition phase.		
5–7	Train one-on-one with trainer in new facility. Focus on how new machines work plus comfort and ecological factors that can influence continued training. Report from any internet access point training data, expectancy rating, and use of any strategies. Enter goals and plan for the next workout.	2	Meet with trainer for orientation session in new facility. Become familiar with environment and equipment. No hands-on training.
8	Train alone in the new facility. Report from any internet access point training data, expectancy rating, and use of any strategies. Receive feedback. Enter goals and plan for the next workout.		
9	Meet with trainer in lab–gym. No training. Review plans and goals for continued training. Review potential barriers and means to make minor protocol changes based on expectancy rating.	3	Meet with trainer in lab–gym. No training. Review progress, comfort level and RT adjustments in new facility.

Author's personal copy

consistent with SCT, problem solving centered on increasing self-efficacy, improving affect, and modifying ecological circumstances, with the overarching goal of facilitating RT maintenance.

To ensure success in transitioning to the new health facility, one of the trainers discussed this decision with each participant and provided a list of approved (by the study PD) community/public health facilities for continued training. The approval process was based on whether the facility had credentialed staff, safety devices (i.e., AED), and RT equipment that was comparable to the machines in the lab–gym and adequate for continued training. As the participants advanced through the transition phase (Table 6), they went from participating in the tracking, recording, and modifying of the RT protocol and ecological circumstances to being responsible for these tasks. Because all training after the initiation phase occurred outside the lab–gym with different RT equipment but all assessments were conducted in the lab–gym with the original RT equipment, it is possible that follow-up assessments underestimated maintenance or increases in strength gains given neuromuscular specificity due to equipment differences [49].

4.1.3. Maintenance phase

The maintenance phase lasted for six months following the transition phase, and the objectives were to consistently train twice per week and continue to attempt to make small strength gains. During this time, participants were expected to enter all training records into the Resist Diabetes website database. There was no face-to-face personal training supervision involved. For the first three months, our follow-up coordinator met one-on-one with participants face-to-face, over the telephone, or via Skype for 15–20 min once every two weeks to review training records, expectancy ratings (to continue RT 2×/wk), and strategies with the goal of making any changes to decrease barriers for training, increase self-efficacy, improve affect, and optimize training. Over the next three months, the meetings occurred once per month, for a total of nine individual sessions. The participants' reported data on the website included exercises performed; repetitions and resistance; form and RPE ratings; an expectancy rating; and answers to a series of simple questions about their ability to plan, schedule, and complete their RT sessions. Our follow-up coordinator also entered notes into each participant's online record tracking the content of each session, including stated goals, progress on goals, and problem solving strategies. The participants received tailored feedback from the study website in written (e.g., suggested strategies to resolve work/training schedule conflicts) and graphic form (e.g., line graphs of strength changes over time), and verbally from the follow-up coordinator, such as on the effectiveness of a problem solving strategy, training sessions planned and completed, days trained, reaching goals in RT, and training at a high RPE.

4.1.4. RT-tracking software

During session 1 of the transition phase, participants created a username- and password-protected study account login which they used to access the Resist Diabetes website during transition and throughout the maintenance and follow-up phases of the study. The website employed software for tracking participants' RT behavior and allowing the participants to schedule RT workouts, develop plans for their workouts, and report workout data. The RT software then generated feedback in graphic form based on each participant's data entries to illustrate RT progress and maintenance.

4.2. Standard care intervention

During the transition phase, the participants randomized to the standard condition followed procedures similar to those used in the SCT condition, but without the hands-on, tailored approach (see Table 6). The participants in the standard group received information in an initial didactic session at the lab–gym about continuing to resistance train and a brief manual on problems frequently encountered and typical solutions. They also received general information about choosing an appropriate facility to continue RT workouts and about local health facilities that had adequate RT equipment, safety procedures, and discounts on membership or fees. Participants also received one orientation session in the new facility with one of the research assistant/personal trainers to become familiar with the environment and the equipment. However, participants did not receive any hands-on training in the new facility.

Standard care participants met with one of the trainers three times during the 4-week transition period. Subsequently, they met with our follow-up coordinator twice during the maintenance phase (once every 8–10 weeks) for didactic sessions primarily involving information about RT, frequently encountered problems, and training strategies after a plateau is reached. The primary purpose of these contacts was to prevent a higher attrition rate in the Standard group and assure safety. There was also continued contact through assessment clinics that are on the same schedule as the SCT group. Although standard participants were expected to access the Resist Diabetes study website, their account capabilities were generic and limited compared to SCT participants. That is, while standard participants could print out and use at their training facility a workout record form that was the same as the SCT condition, they were only able to schedule and report training data and only received generic feedback from the website. They also did not report data on expectancy, answer specific questions, or receive any feedback.

4.3. Follow-up phase

For both the SCT and Standard conditions, contact ended after their respective 6-month maintenance phase which was then followed by a 6-month, no contact follow-up phase.

4.4. RT adherence

At the 9- and 15-month assessment time points, RT adherence was measured with a time-line follow-back approach adapted from prior substance abuse research [50]. On a printed calendar, participants noted each day of RT within the past 30 days. In a given month, 8 sessions (2×/wk) of RT were expected. At each of these assessment points, a participant's score could range from 0 to 8 for RT sessions.

Author's personal copy

5. Data analysis

5.1. Sample size and power calculations

Using a Monte Carlo sample size estimation approach in MPlus [51], we estimated that N = 55 per group would provide sufficient power to detect significant group differences in change over the four assessment points (baseline, 3 months, 9 months and 15 months) for achievement of normal FPG, 2-hr plasma glucose concentration and increases in strength ($\alpha = .05$, effect size = 0.60). The effect size was informed by data from prior studies that do not have a component of RT but have employed similar measures as in the present study [52], as well as from prior studies with RT and T2D [14,15,53–58] that have shown SD estimates of 15% with a maximum difference between placebo and intervention groups to be approximately 15–20% at the final time point, comparable to a medium effect size. For hypothesis 2, we estimated a sample size of 55 per group would provide sufficient power (>.80) to detect a significant medium to large mediation effect size using a regression framework. Testing the mediation model in an SEM framework [59,60], we estimated that a sample of 110 participants could detect a close fitting mediation model. A sample of 170 participants was enrolled to ensure that 110 participants complete all four assessment points of the study, allowing for 35% attrition rate.

5.2. Analysis

We will conduct descriptive univariate analyses on all variables. Data will be examined for outliers, normality and missing data. Violations of normality will be corrected with a transformation procedure. If the proportion of cases with missing data is >5% [61], we will use full-information maximum likelihood (FIML) techniques to address the missing data. This technique is considered to be the most fruitful strategy for recovering unbiased parameter estimates, standard errors and confidence intervals under different conditions of missing data [62]. We will begin by exploring predictors of non-response in order to better understand any discernible systematic processes by using follow-up techniques with participants who drop-out. FIML techniques that make use of all available information will be implemented by software such as STATA and MPlus.

5.2.1. Aim 1 hypothesis analysis plan

The SCT-based intervention will produce significantly greater improvements in markers of prediabetes and in strength than the standard intervention.

We will use growth curve analysis (GCM) to determine the effects of the two intervention modules on prediabetes markers and strength outcomes over time. Since most of the gain occurs in the initial phase, followed by continued growth for the SCT group and likely regression or plateauing for the standard group, there is a potential for a non-linear trend. We will therefore estimate a linear as well as a nonlinear trend by including a polynomial function of time (e.g. time-squared for a quadratic effect) as a covariate to investigate the possibility that a pattern of change other than a linear trajectory could be ascertained. Other non-linear growth models such as a spline or piece-wise models [63] will also be explored in the event the polynomial growth models do not provide a good fit.

Conceptually, GCMs involve estimating individual regressions of the dependent variable over time and adding at the next level predictors of regression parameters of individual trajectories. Each participant's unique trajectory is expressed at level 1 as $y_{it} = \beta_{0i} + \beta_{1i}x_{it} + e_{it}$ where β_{0i} is the intercept for individual *i*, β_{1i} is the slope for the individual *i*, *and* e_{it} is the residual for individual *i* at occasion *t*. The GCM then can yield average values for β_{0i} and β_{1i} (i.e. average fixed effects and average fixed slope), as well as the variance and covariances of these parameters (i.e. random effects). The intercept will be specified at the first occasion of measurement, i.e. stage = 0. Spacing between the repeated measures will be set at 1, 3, and 5 to duplicate the average time of measurement between each occasion. In order to capture the potential nonlinear trajectory after the initiation phase for both of our primary outcomes, our model will estimate a quadratic effect of time by including a polynomial term for time (e.g. time-squared) at Level 1 as a covariate. At Level 2, the GCMs will predict individual differences in the intercept and rate of change as follows:

$$\beta_{0ik} = \gamma_{0k} + \gamma_{01k\,(\text{Intervention group membership})} + \gamma_{02k\,(\text{Person}-\text{level characteristics})} + \mu_{0ik};$$
$$\beta_{1ik} = \gamma_{1k} + \gamma_{10k\,(\text{Intervention group membership})} + \gamma_{11k\,(\text{Person}-\text{level characteristics})} + \mu_{1ik},$$

wherein intervention group status, i.e. SCT vs. standard treatment, will be included to predict individual differences in the intercept and rate of change from intervention group membership (i.e., standard vs. SCT) and person-level control characteristics (e.g., gender, age, BMI). GCMs will be fitted using MPlus. Similar analysis will be repeated for the other secondary outcomes. If deemed necessary, the GCMs will be estimated with a 1000 bootstrapped samples to obtain bias-corrected confidence intervals to compensate for the limited sample size.

5.2.2. Aim 2 hypothesis analysis plan

Positive changes in glucose homeostasis and strength will be mediated by self-efficacy, self-regulation, adherence, and outcome expectancies.

To test the mediation effect of variables such as change in adherence, self-efficacy, self-regulation and OEs over time, we will use two approaches. The aim of a mediation model in regression analysis is to test whether the mediating variable partially or totally accounts for the relationship between the independent and dependent variable [64,65]. Our preliminary analysis would involve estimating the GCMs separately for the mediating variable (MV) and the outcome variable (DV), followed by using the predicted slope parameters derived from these two models as the predictors in the mediation model. If we see any evidence of or trend towards mediation, then we will combine mediation analysis with GCMs [66]. In the mediation model with growth curve analysis, mediation is defined such that the intervention program (IV) influences the growth of the outcome process (DV) indirectly by influencing the growth of the mediator process (MV). Thus, the presence

Author's personal copy

of significant mediation depends on whether the intervention program changes the growth trajectory of the mediator and whether the change of the growth trajectory of the mediator, in turn, is related to the change in the growth trajectory of the outcome variable. First, the growth trajectory of the outcome variable and the growth trajectory of the mediator process will be investigated. Next, the latent growth factors will be regressed on the intervention group membership to account for the program effects, or the mean shift in the growth rate due to the intervention programs. The mediation is then modeled and estimated using a parallel process LGM method [58]. The mediation analysis with latent growth curve analysis will be fitted using MPlus [67]. The fit of the models will be evaluated by using several indices [68], such as the adjusted goodness-of-fit index, the Tucker–Lewis index, standardized root mean square residual and the root mean square error of approximation.

5.3. Data quality/management

Electronic copies of the data are password protected, filed with no identifying information, and stored on computers with firewall protection. The project staff performs all the data management, analysis and archiving tasks. Data entry is computerized and the security of the database is maintained by password-only access, encoding of identifiers to preserve confidentiality, and secure storage of all the assessment measures. The encoded identifier is a randomly generated project assigned number. Links between the encoded identifier and personal participant information (name, address, phone numbers) is stored in a separate, secure location. Procedures for data handling, questionnaire coding, and decision rules for cleaning data have been developed during our previous studies. All data files are encrypted and stored on anti-virus protected computers with firewall protection. The PD is responsible for periodic random checks of entered data.

6. Discussion

Physical activity recommendations for older and diabetic adults from national health organizations, such as ACSM, the American Diabetes Association, American Heart Association, and American Geriatrics Society, include engaging in RT two to three times per week with exercises targeting major muscle groups for a minimum of one set of 8–12 repetitions per exercise [69,70]. However, the National Center for Health Statistics estimated that only ~13% of individuals aged ≥50 years consistently perform RT at least two days per week [71]. Furthermore, this assessment may be an overestimation because RT was defined as performance of 'any strengthening exercise'. Thus, there is a need for more empirically-based interventions to increase RT participation among the aging population.

Preliminary outcomes of the Resist Diabetes study support the initiation and safety of a whole-body RT protocol among older, previously sedentary prediabetic individuals. Participants ($N = 159$) who completed the 3-month initiation phase were adherent (i.e., 91%) to the twice-weekly scheduled RT sessions. The overall low AE rate suggests the effectiveness of conducting initial RT sessions in a controlled lab–gym setting supervised by certified personal trainers. Furthermore, the completion rate of follow-up contacts for the SCT intervention group was acceptable (i.e., 88%) as was attendance for participants at the 9-month (81%) assessment clinics (Note: 15-month assessment clinics in progress), with expected completions providing sufficient power (15 months, >.80). The attendance rate for follow-up contacts and assessment clinics and the AE rate support the feasibility of our RT approach for an older at-risk population.

Of note, our study design and methods are not without limitations. Study recruitment focused on older individuals who were not resistance training and met criteria for prediabetes. Therefore, our results may not extend to young and middle-aged adults, though they also may be sedentary and at a higher risk for developing diabetes. The results from the Resist Diabetes study may have limited generalizability given our enrolled population demographics, with the majority highly educated (high school and beyond), white (93%) individuals (Table 1). The demographics of individuals who were disqualified during the screening process, however, were not different from our enrolled participants with the exception of pre-existing health conditions (i.e., diagnosed heart disease, current diabetes diagnosis, active cancer), medication usage (i.e., <1 year stable dose of BP or cholesterol-lowering meds), and PA levels exceeding minimal study criteria. Our results may not extend to minority groups, as the majority of participants were white. Other factors that may have reduced inclusion and diversity in our study population were study requirements related to Internet access and future health club membership after the supervised training phase to community/public health facilities, although sliding-scale and senior discount rates were available at the local YMCA. The median household income of the Roanoke Valley area is ~$38,000 (2007–2011 US Census Bureau [72]), and some individuals with interest in the project may not have had the financial resources to support in-home internet service or year-long health facility membership fees. We did not supply or support individual internet access, but each study-approved community/public health facility agreed to provide a joining fee waiver or membership fee reduction for our study participants, with monthly fees ranging from $10–$50 as well as a sliding fee scale for low-income individuals at the local YMCA. Thus, affordable options for community/public health facility memberships were available for study participants to relieve these required out-of-pocket expenses.

Additional limitations which should be acknowledged pertain to the use of self-reported methods to assess dietary intake, PA, and RT adherence. Social desirability bias is possible when assessing diet and PA behaviors, and dietary underreporting is a commonly cited limitation when relying on self-reported dietary intake [40]. However, multiple 24-hour recalls are considered superior to other methods for dietary assessment methods, and our preliminary energy intake data appears to be consistent with that reported by nationally representative samples in this age range [41].

Translational behavioral research related to RT initiation and maintenance is lacking evidence from theory-based interventions in adult populations. There are numerous reports from laboratory-controlled RT interventions [7,48,73], but very few studies have examined the outcomes of long-term, less supervised RT in community/public health

Author's personal copy

facilities [54,74,75]. Furthermore, few, if any, studies have employed all SCT constructs (i.e., self-efficacy, self-regulation, OE) to assist individuals overcome barriers to RT in commercial and community facilities during study follow-up periods. The Resist Diabetes study aims to address this theoretical and translational set of issues by demonstrating that a high fidelity SCT-based intervention with extended but faded contact during follow-up may produce greater RT maintenance than more generic, long-term or standard interventions and that SCT variables mediate RT maintenance. Current analysis of our initiation phase outcomes indicate our RT protocol is effective (increased strength), safe (low AE rate), and feasible (acceptable adherence and retention rates) for at-risk adults. However, it is likely that initial RT instruction for at-risk individuals needs to be conducted in a supervised setting, followed by the application of SCT constructs and principles to support transition to and long-term RT maintenance in a community/public health facility.

7. Conclusions

In conclusion, Resist Diabetes is the first long-term randomized controlled trial to apply SCT constructs and strategies in an intervention for initiating and, most importantly, maintaining RT in older adults with prediabetes to improve glucose homeostasis. Unique features of the current study include: the hybrid efficacy/effectiveness approach; a 6-month maintenance intervention phase with faded contact and then a 6-month follow-up phase with no contact after the intervention ends; direct quantitative assessment of fasting glucose and glucose tolerance during an OGTT and estimations of insulin sensitivity, beta-cell responsivity, and disposition index using oral glucose and c-peptide minimal models; application of minimal RT dose following ACSM guidelines (1 set, 8–12 repetitions, multiple muscle exercises, 2×/wk) demonstrating initial efficacy, safety, time efficiency, and adherence; and access to study-specific software for RT tracking via desktop and mobile technology. Findings will provide information on the relationship between the initial 3-month RT protocol and glucose regulation and strength in sedentary prediabetic adults. Significant improvements in the primary and secondary outcome measures for participants randomized to the SCT intervention compared to standard care after the maintenance and follow-up phases will demonstrate the efficacy of SCT constructs and strategies with faded contact on adherence to minimally supervised RT. If successful, this trial may provide evidence for using long-term theory-based interventions with RT in future investigations and translational efforts for diabetes and disease prevention programs in the aging population.

Funding

This study was funded by National Institutes of Health grant R01 DK082383.

Acknowledgments

We would like to thank Sheila Winett MS, Dave Williams PhD, John Pownall RN, Mary Elizabeth Baugh MS RD, Kyle Flack, Nabil Boutagy MS, Daniel Gochenour, Tanya Halliday RD, Adrienne Clark MS, Valisa Hedrick PhD RD, Rachel Cornett MS RD, Soheir Boshra MD, and Joshua Eikenberg MPH.

References

[1] Centers for Disease Control, Prevention. National diabetes fact sheet: national estimates and general information on diabetes and prediabetes in the United States, 2011. Atlanta, GA: U.S. Department of Health and Human Services, Centers for Disease Control and Prevention; 2011.
[2] Nathan DM, Davidson MB, DeFronzo RA, Heine RJ, Henry RR, Pratley R, et al. Impaired fasting glucose and impaired glucose tolerance: implications for care. Diabetes Care 2007;30:753–9.
[3] American College of Sports Medicine Position Stand. Exercise and physical activity for older adults. Med Sci Sports Exerc 1998;30: 992–1008.
[4] American College of Sports Medicine. ACSM's guidelines for exercise testing and prescription. 7th ed. Philadelphia: Lippincott, Williams & Wilkins; 2005.
[5] Hurley BF, Roth SM. Strength training in the elderly: effects on risk factors for age-related diseases. Sports Med 2000;30:249–68.
[6] Winett RA, Carpinelli RN. Potential health-related benefits of resistance training. Prev Med 2001;33:503–13.
[7] Seguin R, Nelson ME. The benefits of strength training for older adults. Am J Prev Med 2003;25:141–9.
[8] Eves ND, Plotnikoff RC. Resistance training and type 2 diabetes: considerations for implementation at the population level. Diabetes Care 2006;29:1933–41.
[9] Eriksson J, Taimela S, Eriksson K, Parviainen S, Peltonen J, Kujala U. Resistance training in the treatment of non-insulin-dependent diabetes mellitus. Int J Sports Med 1997;18:242–6.
[10] Poehlman ET, Dvorak RV, DeNino WF, Brochu M, Ades PA. Effects of resistance training and endurance training on insulin sensitivity in nonobese, young women: a controlled randomized trial. J Clin Endocrinol Metab 2000;85:2463–8.
[11] Miller WJ, Sherman WM, Ivy JL. Effect of strength training on glucose tolerance and post-glucose insulin response. Med Sci Sports Exerc 1984;16:539–43.
[12] Holten MK, Zacho M, Gaster M, Juel C, Wojtaszewski JF, Dela F. Strength training increases insulin-mediated glucose uptake, GLUT4 content, and insulin signaling in skeletal muscle in patients with type 2 diabetes. Diabetes 2004;53:294–305.
[13] Eriksson J, Tuominen J, Valle T, Sundberg S, Sovijarvi A, Lindholm H, et al. Aerobic endurance exercise or circuit-type resistance training for individuals with impaired glucose tolerance? Horm Metab Res 1998;30:37–41.
[14] Ishii T, Yamakita T, Sato T, Tanaka S, Fujii S. Resistance training improves insulin sensitivity in NIDDM subjects without altering maximal oxygen uptake. Diabetes Care 1998;21:1353–5.
[15] Castaneda C, Layne JE, Munoz-Orians L, Gordon PL, Walsmith J, Foldvari M, et al. A randomized controlled trial of resistance exercise training to improve glycemic control in older adults with type 2 diabetes. Diabetes Care 2002;25:2335–41.
[16] Shimokata H, Muller DC, Fleg JL, Sorkin J, Ziemba AW, Andres R. Age as independent determinant of glucose tolerance. Diabetes 1991;40: 44–51.
[17] Short KR, Nair KS. Muscle protein metabolism and the sarcopenia of aging. Int J Sport Nutr Exerc Metab 2001;11:S119–27 [Suppl.].
[18] Nelson ME, Rejeski WJ, Blair SN, Duncan PW, Judge JO, King AC, et al. Physical activity and public health in older adults: recommendation from the American College of Sports Medicine and the American Heart Association. Circulation 2007;116:1094–105.
[19] Winett RA, Williams DM, Davy BM. Initiating and maintaining resistance training in older adults: a social cognitive theory-based approach. Br J Sports Med 2009;43:114–9.
[20] Engelgau MM, Vinicor F, Simionescu M, King GL, Meininger C, Mensah GA. Summary statement IV: obesity and diabetes: opportunities for translation of basic research. Vascul Pharmacol 2007;46:324–6.
[21] Buchanan TA. (How) can we prevent type 2 diabetes? Diabetes 2007;56:1502–7.
[22] Ackermann RT, Finch EA, Brizendine E, Zhou H, Marrero DG. Translating the diabetes prevention program into the community. The DEPLOY pilot study. Am J Prev Med 2008;35:357–63.
[23] Estabrooks PA, Smith-Ray RL. Piloting a behavioral intervention delivered through interactive voice response telephone messages to promote weight loss in a pre-diabetic population. Patient Educ Couns 2008;72:34–41.

Author's personal copy

[24] Boltri JM, Davis-Smith YM, Seale JP, Shellenberger S, Okosun IS, Cornelius ME. Diabetes prevention in a faith-based setting: results of translational research. J Public Health Manag Pract 2008;14:29–32.
[25] Amundson HA, Butcher MK, Gohdes D, Hall TO, Harwell TS, Helgerson SD, et al. Translating the diabetes prevention program into practice in the general community: findings from the Montana Cardiovascular Disease and Diabetes Prevention Program. Diabetes Educ 2009;35:209–10 [13–4, 16–20].
[26] McTigue KM, Conroy MB, Bigi L, Murphy C, McNeil M. Weight loss through living well: translating an effective lifestyle intervention into clinical practice. Diabetes Educ 2009;35(8):199–204.
[27] Vinicor F. Invited commentary: translating diabetes research. Arch Intern Med 2008;168:199.
[28] The Diabetes Prevention Program. Design and methods for a clinical trial in the prevention of type 2 diabetes. Diabetes Care 1999;22:623–34.
[29] American College of Sports Medicine Position Stand and American Heart Association. Recommendations for cardiovascular screening, staffing, and emergency policies at health/fitness facilities. Med Sci Sports Exerc 1998;30:1009–18.
[30] Fletcher GF, Balady GJ, Amsterdam EA, Chaitman B, Eckel R, Fleg J, et al. Exercise standards for testing and training: a statement for healthcare professionals from the American Heart Association. Circulation 2001;104:1694–740.
[31] National High Blood Pressure Education Program. The seventh report of the Joint National Committee on prevention, detection, evaluation, and treatment of high blood pressure. Bethesda, MD: National Heart, Lung, and Blood Institute (US); 2004.
[32] Perloff D, Grim C, Flack J, Frohlich ED, Hill M, McDonald M, et al. Human blood pressure determination by sphygmomanometry. Circulation 1993;88:2460–70.
[33] Chobanian AV, Bakris GL, Black HR, Cushman WC, Green LA, Izzo Jr JL, et al. Seventh report of the Joint National Committee on Prevention, Detection, Evaluation, and Treatment of High Blood Pressure. Hypertension 2003;42:1206–52.
[34] American Diabetes Association. Diagnosis and classification of diabetes mellitus. Diabetes Care 2007;30(Suppl. 1):S42–7.
[35] Dalla Man C, Campioni M, Polonsky KS, Basu R, Rizza RA, Toffolo G, et al. Two-hour seven-sample oral glucose tolerance test and meal protocol: minimal model assessment of beta-cell responsivity and insulin sensitivity in nondiabetic individuals. Diabetes 2005;54:3265–73.
[36] Dalla Man C, Caumo A, Basu R, Rizza R, Toffolo G, Cobelli C. Minimal model estimation of glucose absorption and insulin sensitivity from oral test: validation with a tracer method. Am J Physiol Endocrinol Metab 2004;287:E637–43.
[37] Dalla Man C, Caumo A, Cobelli C. The oral glucose minimal model: estimation of insulin sensitivity from a meal test. IEEE Trans Biomed Eng 2002;49:419–29.
[38] Dalla Man C, Yarasheski KE, Caumo A, Robertson H, Toffolo G, Polonsky KS, et al. Insulin sensitivity by oral glucose minimal models: validation against clamp. Am J Physiol Endocrinol Metab 2005;289:E954–9.
[39] Breda E, Cavaghan MK, Toffolo G, Polonsky KS, Cobelli C. Oral glucose tolerance test minimal model indexes of beta-cell function and insulin sensitivity. Diabetes 2001;50:150–8.
[40] Thompson FE, Subar AF. Dietary assessment methodology. Nutrition in the prevention and treatment of disease. 3rd ed. Academic Press, Elsevier, Inc.; 2013. p. 5–16.
[41] Ford ES, Dietz WH. Trends in energy intake among adults in the United States: findings from NHANES. Am J Clin Nutr 2013;97:848–53.
[42] Noble BJ, Borg GA, Jacobs I, Ceci R, Kaiser P. A category-ratio perceived exertion scale: relationship to blood and muscle lactates and heart rate. Med Sci Sports Exerc 1983;15:523–8.
[43] The Aerobics Center longitudinal study physical activity questionnaire. Med Sci Sports Exerc 1997;29:10–4.
[44] Anderson-Bill ES, Winett RA, Wojcik JR, Winett SG. Web-based guide to health: relationship of theoretical variables to change in physical activity, nutrition and weight at 16-months. J Med Internet Res 2011;13:e27.
[45] http://www.iom.edu/Activities/Nutrition/SummaryDRIs/DRI-Tables.aspx; July 11 2013.
[46] U.S. Department of Agriculture and U.S. Department of Health and Human Services. 7th ed. Washington, D.C.: U.S. Government Printing Office; 2010
[47] Burd NA, West DW, Staples AW, Atherton PJ, Baker JM, Moore DR, et al. Low-load high volume resistance exercise stimulates muscle protein synthesis more than high-load low volume resistance exercise in young men. PLoS One 2010;5:e12033.
[48] Phillips SM, Winett RA. Uncomplicated resistance training and health-related outcomes: evidence for a public health mandate. Curr Sports Med Rep 2010;9:208–13.
[49] Mitchell CJ, Churchward-Venne TA, West DW, Burd NA, Breen L, Baker SK, et al. Resistance exercise load does not determine training-mediated hypertrophic gains in young men. J Appl Physiol 2012;113:71–7.
[50] Sobell LC, Sobell MB. Alcohol timeline followback (TLFB). Handbook of psychiatric measures. Washington, D.C.: American Psychiatric Association; 2000. p. 477–9.
[51] Muthén LK, Muthén BO. How to use a Monte Carlo study to decide on sample size and determine power. Struct Equ Model 2002;4:599–620.
[52] Knowler WC, Barrett-Connor E, Fowler SE, Hamman RF, Lachin JM, Walker EA, et al. Reduction in the incidence of type 2 diabetes with lifestyle intervention or metformin. N Engl J Med 2002;346:393–403.
[53] Ibanez J, Izquierdo M, Arguelles I, Forga L, Larrion JL, Garcia-Unciti M, et al. Twice-weekly progressive resistance training decreases abdominal fat and improves insulin sensitivity in older men with type 2 diabetes. Diabetes Care 2005;28:662–7.
[54] Dunstan DW, Vulikh E, Owen N, Jolley D, Shaw J, Zimmet P. Community center-based resistance training for the maintenance of glycemic control in adults with type 2 diabetes. Diabetes Care 2006;29:2586–91.
[55] Dunstan DW, Daly RM, Owen N, Jolley D, De Courten M, Shaw J, et al. High-intensity resistance training improves glycemic control in older patients with type 2 diabetes. Diabetes Care 2002;25:1729–36.
[56] Dunstan DW, Puddey IB, Beilin LJ, Burke V, Morton AR, Stanton KG. Effects of a short-term circuit weight training program on glycaemic control in NIDDM. Diabetes Res Clin Pract 1998;40:53–61.
[57] Fenicchia LM, Kanaley JA, Azevedo Jr JL, Miller CS, Weinstock RS, Carhart RL, et al. Influence of resistance exercise training on glucose control in women with type 2 diabetes. Metabolism 2004;53:284–9.
[58] Kraemer WJ, Adams K, Cafarelli E, Dudley GA, Dooly C, Feigenbaum MS, et al. American College of Sports Medicine position stand. Progression models in resistance training for healthy adults. Med Sci Sports Exerc 2002;34:364–80.
[59] MacCallum RC, Browne MW, Sugawara HM. Power analysis and determination of sample size for covariance structure modeling. Psychol Methods 1996;1:130–49.
[60] Satorra A, Saris WE. Power of the likelihood ratio test in covariance structure analysis. Psychometrika 1985;50:83–90.
[61] Little RJA, Rubin DB. Statistical analysis with missing data. 2nd ed. Hoboken, NJ: Wiley; 2002.
[62] Schafer JL, Graham JW. Missing data: our view of the state of the art. Psychol Methods 2002;7:147–77.
[63] Singer JD, Willett JB. Applied longitudinal data analysis: modeling changes and event occurrence. New York: Oxford University Press; 2003.
[64] Baron RM, Kenny DA. The moderator–mediator variable distinction in social psychological research: conceptual, strategic, and statistical considerations. J Pers Soc Psychol 1986;51:1173–82.
[65] MacKinnon DP, Dwyer JH. Estimating mediated effects in prevention studies. Eval Rev 1993;17:144–58.
[66] Cheong J, MacKinnon DP, Khoo ST. A latent growth modeling approach to mediation analysis [Abstract]. New methods for the analysis of change. Washington, D.C.: American Psychological Association; 2001. p. 390–2.
[67] Muthén BO, Muthén LK. Mplus user's guide. 6th ed. Los Angeles, CA: Muthén & Muthén; 1998–2010.
[68] Davey A, Savla JS, Luo Z. Issues in evaluation model fit with missing data. Struct Equ Model 2005;12:578–97.
[69] Sigal RJ, Kenny GP, Wasserman DH, Castaneda-Sceppa C, White RD. Physical activity/exercise and type 2 diabetes: a consensus statement from the American Diabetes Association. Diabetes Care 2006;29:1433–8.
[70] Chodzko-Zajko WJ, Proctor DN, Fiatarone Singh MA, Minson CT, Nigg CR, Salem GJ, et al. American College of Sports Medicine position stand. Exercise and physical activity for older adults. Med Sci Sports Exerc 2009;41:1510–30.
[71] Kruger J, Carlson SA, Buchner D. How active are older Americans? Prev Chronic Dis 2007;4:A53.
[72] http://quickfacts.census.gov/qfd/states/51/5168000.html; October 24 2013.
[73] Ratamess NA. American College of Sports Medicine position stand. Progression models in resistance training for healthy adults. Med Sci Sports Exerc 2009;41:687–708.
[74] Dunstan DW, Daly RM, Owen N, Jolley D, Vulikh E, Shaw J, et al. Home-based resistance training is not sufficient to maintain improved glycemic control following supervised training in older individuals with type 2 diabetes. Diabetes Care 2005;28:3–9.
[75] Schmitz KH, Hannan PJ, Stovitz SD, Bryan CJ, Warren M, Jensen MD. Strength training and adiposity in premenopausal women: strong, healthy, and empowered study. Am J Clin Nutr 2007;86:566–72.
